# Nerve growth factor modulates the tumor cells migration in ovarian cancer through the WNT/β-catenin pathway

**DOI:** 10.18632/oncotarget.13186

**Published:** 2016-11-07

**Authors:** Bo Li, Shaoxi Cai, Yi Zhao, Qiyi He, Xiaodong Yu, Longcong Cheng, Yingfeng Zhang, Xiancheng Hu, Ming Ke, Sijia Chen, Misha Zou

**Affiliations:** ^1^ Key laboratory of Biorheological Science and Technology, Ministry of Education, College of Bioengineering, Chongqing University, Chongqing, China; ^2^ School of Education, Chongqing Normal University, Chongqing, China; ^3^ College of Life Sciences, Chongqing Normal University, Chongqing, China; ^4^ College of Chemistry, Chongqing Normal University, Chongqing, China

**Keywords:** NGF, NGFRs, WNT/β-catenin pathway, 3D microfluidic device, migration

## Abstract

Nerve growth factor (NGF)/nerve growth factor receptors (NGFRs) axis and canonical WNT/β-catenin pathway have shown to play crucial roles in tumor initiation, progression and prognosis. But little did we know the relationship between them in modulation of tumor progress. In this report, we found that NGF/NGFRs and β-catenin were coexpression in ovarian cancer cell lines, and NGF can decrease the expression level of β-catenin and affect its activities, which may be related to the NGF-induced down-regulation of B-cell CLL/lymphoma 9-like (BCL9L, BCL9-2). Furthermore, NGF can also increase or decrease the downstream target gene expression levels of WNT/β-catenin depending on the cell types. Especially, we created a novel *in vitro* cell growth model based on a microfluidic device to intuitively observe the effects of NGF/NGFRs on the motility behaviors of ovarian cancer cells. The results showed that the migration area and maximum distance into three dimensional (3D) matrigel were decreased in CAOV3 and OVCAR3 cells, but increased in SKOV3 cells following the stimulation with NGF. In addition, we found that the cell colony area was down-regulated in CAOV3 cells, however, it was augmented in OVCAR3 cells after treatment with NGF. The inhibitors of NGF/NGFRs, such as Ro 08-2750, K252a and LM11A-31,can all block NGF-stimulated changes of gene expression or migratory behavior on ovarian cancer cells. The different results among ovarian cancer cells illustrated the heterogeneity and complexity of ovarian cancer. Collectively, our results suggested for the first time that NGF is functionally linked to β-catenin in the migration of human ovarian cancer cells, which may be a novel therapeutic perspective to prevent the spread of ovarian carcinomas by studying the interaction between NGF/NGFRs and canonical WNT/β-catenin signaling.

## INTRODUCTION

Ovarian cancer is the most lethal gynecologic malignancy, and each year more than 200,000 women are diagnosed with ovarian cancer, and about 100,000 patients die of its complications in worldwide [1]. Despite surgical reduction and platinum-based systemic treatment have achieved great progress, the majority of patients die from the metastasis of ovarian cancer cells and disease recurrence [2, 3]. The etiology and pathology of ovarian cancer are very complicated and not adequately understood, although the ovarian surface epithelial-mesenchymal “metamorphosis” hypothesis during ovarian carcinoma metastasis has gained acceptance widely [4–6]. Clinically, ovarian carcinoma is easy to form malignant ascites and diffuse multi-focal intraperitoneal metastasis [7, 8]. The unique metastatic niche in intraperitoneal dissemination formed by growth factors, chemokines, extracellular matrix proteins, pro-inflammatory cytokines, and so on released by both tumor or host cells within the ovarian carcinoma microenvironment, which constitutes an integrated pathologic network and provides ample opportunity for signaling cross-talk among several major signaling pathways involved them to modify the clinical outcome of the disease [9–13].

NGF is a typical prototypic compound of neurotrophins which belong to the family of growth factors. NGF exerts its biological effects through two cognate receptors on target cell surface: TrkA and P75. TrkA is a high-affinity tyrosine kinase receptor which possesses a tyrosine kinase catalytic domain and mainly mediates the trophic effects of NGF. P75 is a low-affinity, non-selective neurotrophin receptor of the tumor necrosis receptor superfamily, which lacks intrinsic catalytic activity and is able to bind all neurotrophins with approximately equal affinity [14–16]. TrkA and P75 collaborate with each other at the plasma membrane to bind NGF, but seem to have an antagonistic relationship in other ways which can activate different cellular responses under a variety of conditions by different mechanisms [17, 18]. In early years, the attention of NGF was mainly focused on cell survival, proliferation, differentiation, angiogenesis, and so on in nervous or non-nervous system [19–23]. During recent years, a growing body of evidence supports a role for NGF/NGFRs signaling in tumorigenesis and progression which can urged cancer cells to override normal cell growth regulatory mechanisms [24–26]. The abnormal expression of NGF/NGFRs may alter cell death and survival, cell proliferation, invasion and metastasis in various cancer cells depending on cell types, the receptor types, the expression levels of receptors or adaptor proteins *via* activation and regulation of a variety of signaling pathways, such as NF-κB, PI3K/Akt, Ras/MAPK, and so forth [27–31].

WNT signaling pathways, including canonical (WNT/β-catenin) and non-canonical pathways, play crucial roles in maintaining homeostasis of a variety of tissues and regulating morphology, survival/apoptosis, proliferation, differentiation, polarity, adhesion, motility and other important cellular processes in physiology and pathology situations [32–35]. The provoked canonical WNT/β-catenin signaling pathway can regulate the expression levels of a number of genes in bone diseases, cardiovascular diseases and cancers [36–39]. The emerging data have shown that NGF/NGFRs are overexpressed in ovarian cancer tissues and cells, but very low levels in normal ovarian tissues, which are correlated with the initiation, progression and prognosis of human ovarian cancers [25, 40]. And the WNT/β-catenin pathway plays an important role in carcinogenesis and development of all ovarian cancer subtypes [41–43]. However, there are few studies to document the relationship and exact molecular mechanism between NGF signaling and WNT/β-catenin signaling, two important molecular signaling pathways, in modulating the invasion and migration of ovarian cancer cells.

In this study, we investigated the expression of NGF/NGFRs and β-catenin in ovarian cancer cells. NGF acted as an autocrine or paracrine regulator of β-catenin which can decrease β-catenin expression and affect the activation status of β-catenin in ovarian cancer cells. Some specific antagonists, such as Ro 08-2750 (to NGF), K252a (to TrkA) or LM11A-31 (to P75), can all increase β-catenin expression by inhibiting the roles of NGF/NGFRs in ovarian cancer cells. A meaningful findings was that B-cell CLL/lymphoma 9-like (BCL9L, BCL9-2), a control switch for regulating β-catenin, was decreased after treatment with NGF. Our results confirmed that NGF may affect the expression or activity of β-catenin by regulating the expression levels of BCL9-2 in ovarian cancer cells. Next, we assessed the effects of NGF/NGFRs on the expression of several downstream target genes related WNT/β-catenin, such as cluster of differentiation 44 (CD44), cellular homologue of avian myelocytomatosis virus oncogene (C-myc), matrix metalloproteinase 2 (MMP2), matrix metalloproteinase 7(MMP7) and tissue inhibitors of metalloproteinase 2 (TIMP2), which had revealed significant changes in ovarian cancer cells, and the inhibition of NGF, TrkA or P75 can reverse their expressions. In addition, we used transwell assay, especially, 3D microfluidic chip experiment which is a novel *in vitro* cell growth model based on a microfluidic device, to obtain more intuitive experimental data of migration stimulated with NGF and NGF/NGFRs-related inhibitors. We observed that NGF can affect the cell motility and migration ability. Altogether, the results presented here show that NGF may function as a mediator of ovarian cancer cell growth and migration by modulating canonical WNT/β-catenin signaling pathway.

## RESULTS

### NGFRs and endogenous NGF expression in ovarian cancer cells

**Figure 1:**
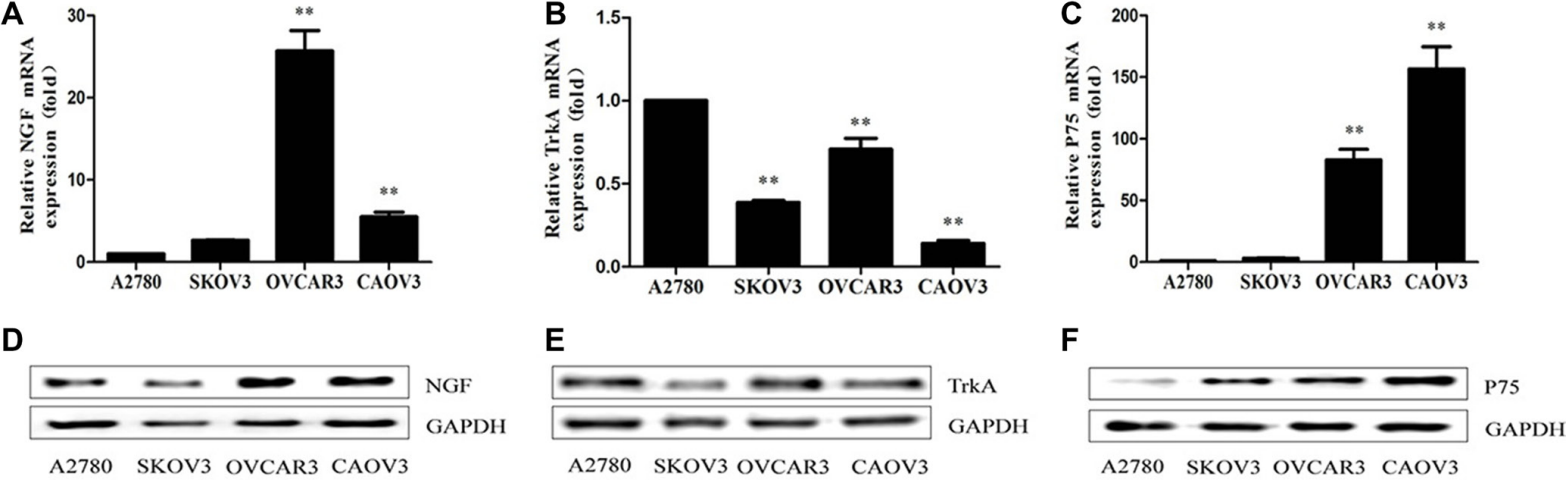
NGF/NGFRs expression in human ovarian cancer cells (**A**–**C**) NGF, TrkA and P75 mRNA expression in ovarian cancer cells under the normal cultured condition measured by qPCR analysis. (**D**–**F**) NGF, TrkA and P75 protein expression in ovarian cancer cells measured by western blot with the indicated antibodies. The relative mRNA expression was normalized against GAPDH mRNA. The value are expressed as mean ± sd. compared with A2780 (**p < 0.01). GAPDH protein expression served as a control for western blot. Data represent three replicate experiments, independently.

Previous findings showed that NGF generated intracellular signals by interacting with its membrane receptors. NGF/NGFRs complex can stimulate cellular proliferation of human ovarian cancer cells and participate in extracellular-matrix remodeling, formation of novel blood-vessels, pathological angiogenic processes and so on that affect the migratory behavior of ovarian cancer cells [44–46]. Here, we determined the expression of NGFRs and endogenous NGF in the four different epithelial ovarian cancer cell lines by quantitative real-time PCR (qPCR) and western blot. Variable levels of NGFRs and endogenous NGF were observed in the four ovarian cancer cells. Endogenous NGF was found lower expression in A2780 and SKOV3 cells, and higher expression was shown in OVCAR3 and CAOV3 (Figure 1A, 1D; Supplementary Figure S1A), which suggested that NGF may regulate the biological behaviors of ovarian cancer cells through an autocrine loop. A low-level expression of TrkA was detected in SKOV3 and CAOV3 cells, but a higher-level expression of TrkA was present in A2780 and OVCAR3 cells (Figure 1B, 1E; Supplementary Figure S1B). TrkA shows to mainly mediate the trophic effects of NGF, and the overexpression, rearrangement or mutation of the TrkA gene can result in constitutive activation of the receptor, which increased the tumorigenicity and tumor progression [47–51]. A weaker expression of P75 was observed in A2780 and SKOV3 cells compared to other two ovarian cancer cell lines (Figure 1C, 1F; Supplementary Figure S1C). The expression of P75 can act as a bifunctional switch directing the cell down opposing paths of cell death or survival, and regulate the growth and metastasis of cancer cells [52–56]. The differential expression levels of NGF/NGFRs in ovarian cancer cells suggest that they may have some functional roles in the occurrence and progression in ovarian cancer.

### Expression of β-catenin in ovarian cancer cells

**Figure 2:**
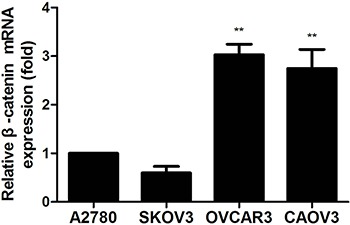
The β-catenin mRNA expression in human ovarian cancer cells measured by qPCR analysis The mRNA expression of β-catenin was observed in ovarian cancer cells under the normal cultured condition. The relative mRNA expression was normalized against GAPDH mRNA. The mRNA value represents fold difference compared to A2780 group (**p < 0.01). Representative of three independent experiments.

β-catenin is a critical effector of the highly conserved canonical WNT signaling pathway which can control both cadherin-mediated cell adhesion and activation of WNT target genes [57, 58]. It has been described that in many normal cells, β-catenin binds to APC, GSK3β or Axin which leads to degradation of β-catenin through the ubiquitin-proteasome pathway, the levels of β-catenin are maintained at low concentrations in the cytoplasm and nuclear. Inappropriate activation of WNT signaling leads to functional disruption of the β-catenin degradation complex and accumulation of free pool of β-catenin in cytoplasm or nucleus where the unphosphorylated (“activated”) β-catenin promotes the transcription of specific WNT target genes by binding to TCF transcription factors in the nucleus, which are the basis for WNT-induced changes in cancer progression and development [59–67]. The aberrant expression of β-catenin gene (CTNNB1) is commonly observed in ovarian carcinoma, it is an important molecular marker which promotes ovarian cancer progression via diverse mechanisms including gene mutations or non-genetic mutation fashions in the presence of extracellular inhibitors and intranuclear transcription cofactors [68–70]. In this study, we found that a weak mRNA expression of β-catenin was detected in A2780 and SKOV3 cells, whereas the mRNA expression of β-catenin was remarkably higher in OVCAR3 and CAOV3 cells compared with the first two kinds of cells (Figure 2). The mRNA expression of β-catenin in SKOV3, OVCAR3 and CAOV3 was related to endogenous levels of NGF.

### NGF regulates the expression levels of β-catenin in ovarian cancer cells

**Figure 3:**
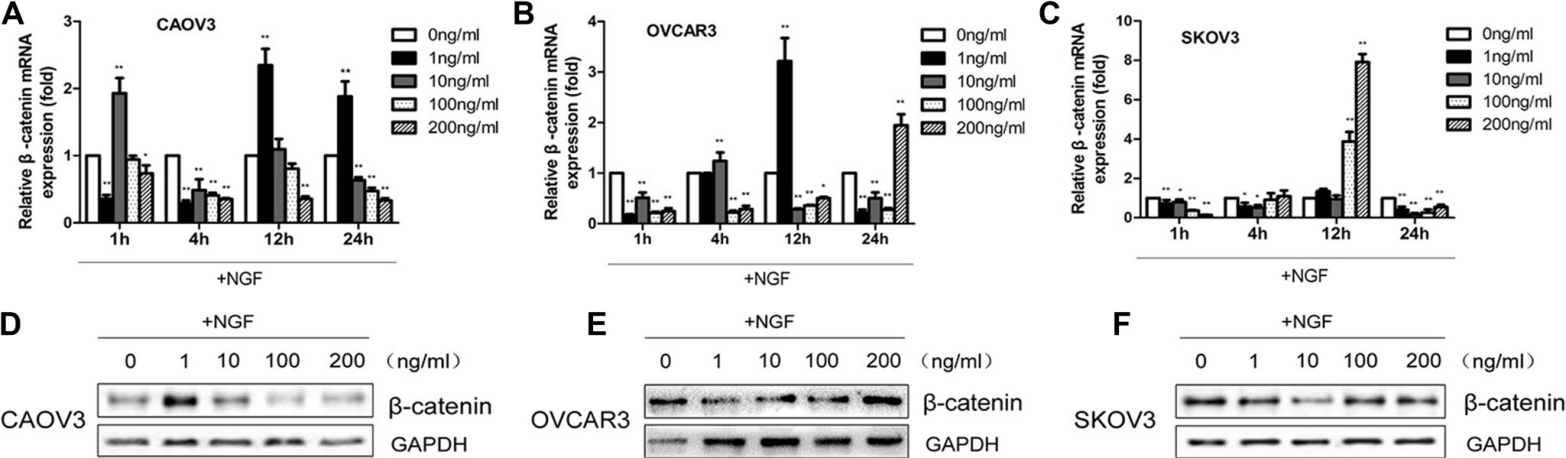
The β-catenin mRNA and protein expression in ovarian cancer cells stimulated with different concentrations of recombinant human β-NGF (**A**–**C**) The changes of β-catenin mRNA expression in CAOV3, OVCAR3 and SKOV3 cells following stimulation with 0, 1, 10, 100 and 200 ng/ml NGF at indicated time points by qPCR analysis. The relative mRNA expression was normalized against GAPDH mRNA levels. The values are expressed as mean ± sd. and represented fold difference in relation to the 0 ng/ml NGF group (*p < 0.05, **p < 0.01). The results are representative of three replicate experiments. (D–F) The β-catenin protein expression levels were analyzed in CAOV3, OVCAR3 and SKOV3 cells treated with different concentrations of recombinant human β-NGF (0–200 ng/ml) at 24 hour time point by western blot. GAPDH expression served as control. Data represent one of three replicate studies.

To investigate whether NGF is involved in the regulation of WNT/β-catenin signaling pathway in ovarian cancer and the effects in response to NGF are autocrine or paracrine fashion, SKOV3, OVCAR3 and CAOV3 were chosen to be as following experimental materials for higher endogenous NGF expression levels. We treated the three cells with 0, 1, 10, 100 and 200 ng/ml recombinant human β-NGF in serum-free medium and sustained for 24 hours. The results revealed that the changes of β-catenin mRNA expression were no consistency and stability following the stimulation of different concentrations of NGF at 1, 4 and 12 hour time point, but to be sure that NGF can lead to abnormal expression of β-catenin in the three ovarian cancer cell lines. At 24 hour time point, the maximal down-regulation was observed in all of the three cells when stimulated with 100 ng/ml NGF (Figure 3A–3C). We next examined the protein expression of β-catenin in the three cells at 24 hour, and the results showed down-regulation of β-catenin after treatment with 100 ng/ml NGF (Figure 3D–3F; Supplementary Figure S2A–2C), consistent with the changes of mRNA. Our results indicated that exogenous NGF can down-regulate the expression levels of β-catenin in ovarian cancer cells.

### The effect of NGF/NGFRs-related inhibitors on β-catenin expression in ovarian cancer cells

**Figure 4:**
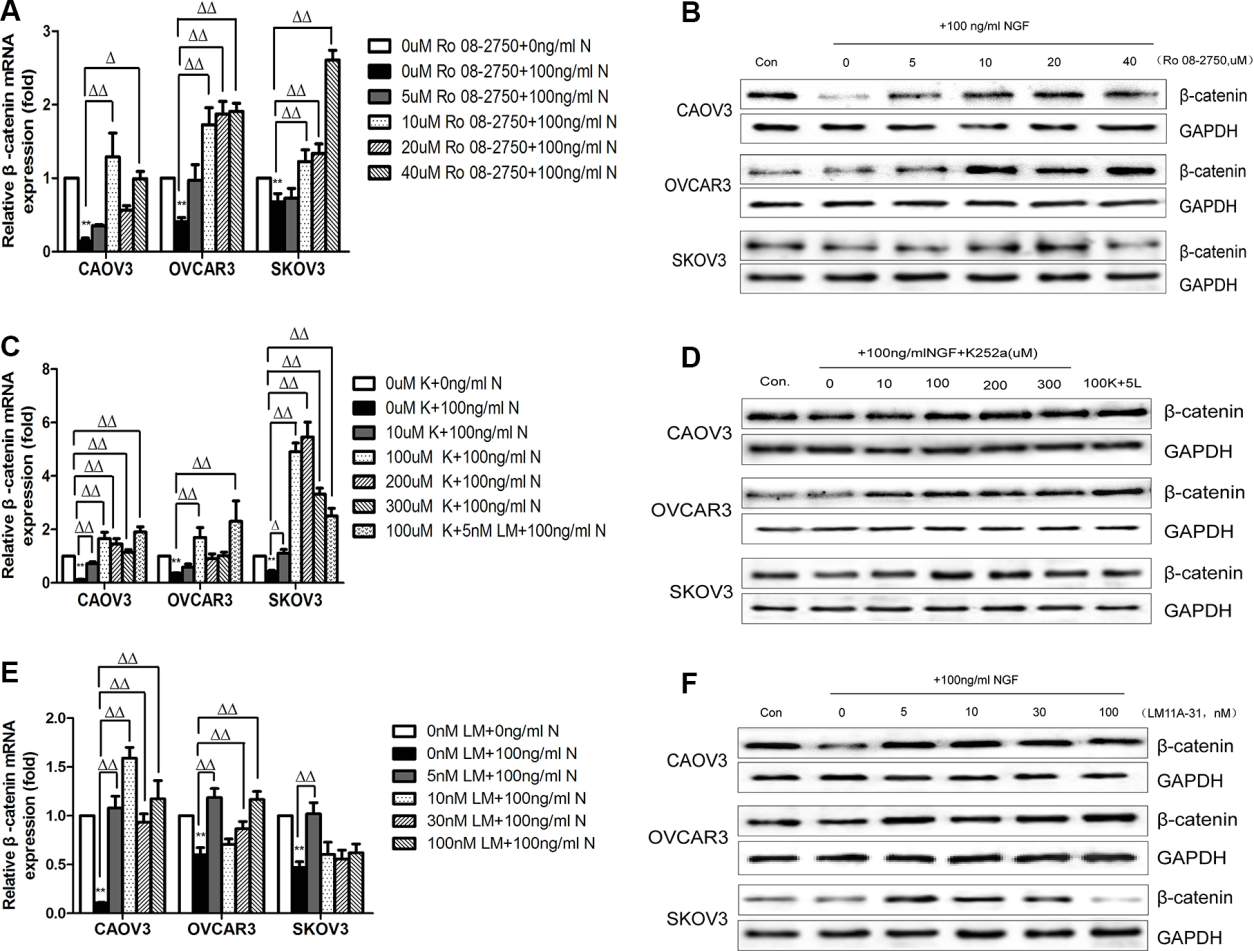
The effect of NGF/NGFRs-related inhibitors on β-catenin expression in ovarian cancer cells at 24 hour time point (**A**) The β-catenin mRNA expression in ovarian cancer cells treated with different concentrations of Ro 08-2750. The mRNA value represents fold difference compared to the untreated control (0 uM Ro 08-2750+0 ng/ml NGF, **p < 0.01) and 0 uM Ro 08-2750+100 ng/ml NGF group (Δ*p* < 0.05, ΔΔ*p* < 0.01). (**B**) Total cell protein lysates were analyzed for β-catenin expression by western blot. (Con: 0 uM Ro 08-2750+0 ng/ml NGF; 0: 0 uM Ro 08-2750+100 ng/ml NGF; 5: 5 uM Ro 08-2750+100 ng/ml NGF; 10: 10 uM Ro 08-2750+100 ng/ml NGF; 20: 20 uM Ro 08-2750+100 ng/ml NGF; 40: 40 uM Ro 08-2750+100 ng/ml NGF). (**C**) The β-catenin mRNA expression in ovarian cancer cells treated with different concentrations of K252a. The mRNA value represents fold difference compared to the untreated control (0 uM K252a+0 ng/ml NGF, **p < 0.01) and 0 uM K252a+100 ng/ml NGF group (Δ*p* < 0.05, ΔΔ*p* < 0.01). (**D**) Total cell protein lysates were detected for β-catenin expression by western blot. (Con: 0 uM K252a+0 ng/ml NGF; 0: 0 uM K252a+100 ng/ml NGF; 10: 10 uM K252a+ 100 ng/ml NGF; 100: 100 uM K252a+100 ng/ml NGF; 200: 200 uM K252a+100 ng/ml NGF; 300:300 uMK252a+100 ng/ml NGF; 100K+5L: 100 uM K252a+5 nM LM11A-31+100 ng/ml NGF). (**E**) The β-catenin mRNA expression in ovarian cancer cells treated with different concentrations of LM11A-31. The mRNA value represents fold difference compared to the untreated control (0 nM LM11A-31+0 ng/ml NGF, **p < 0.01) and 0 nM LM11A-31+100 ng/ml NGF group (ΔΔ*p* < 0.01). (**F**) Total cell protein lysates were analyzed for β-catenin expression by western blot. (Con: 0 nM LM11A-31+0 ng/ml NGF; 0: 0 nM LM11A-31+100 ng/ml NGF; 5: 5 nM LM11A-31+100 ng/ml NGF; 10: 10 nM LM11A-31+100 ng/ml NGF; 30: 30 nM LM11A-31+100 ng/ml NGF; 100: 100 nM LM11A-31+100 ng/ml NGF). GAPDH expression served as control. All of the data and figures are representative of three independent replicate studies.

In previous studies, we knew that the expression of β-catenin was reduced following the stimulation with 100 ng/ml NGF at 24 hour time point. To further understand the role of NGF on cellular levels of β-catenin, CAOV3, OVCAR3 and SKOV3 were treated with NGF/NGFRs-related inhibitors and sustained for 24 hour observation. At the time point of 24 hour, mRNA and protein expression levels of β-catenin were determined by qPCR and western blot. In the present study, we investigated the effect of 0, 5, 10, 20 and 40 uM NGF-related inhibitor Ro 08-2750; 0, 10, 100, 200 and 300 uM TrkA-related inhibitor K252a; and 0, 5, 10, 30 and 100 nM P75-related inhibitor LM11A-31 on the gene expression changes of β-catenin in ovarian cancer cells. Ro 08-2750, a small non-peptide molecule, was found to bind to NGF and dimer itself, which induces a concentration-dependent and time-dependent conformational change of NGF that depending on the cell types, cell growth conditions or combination form with receptors. At low concentrations of Ro 08-2750, NGF can no longer bind to P75, whereas binding to TrkA is not impaired. Higher concentrations of Ro 08-2750, the NGF/Ro 08-2750 complex did not bind to TrkA as well, which is a powerful tool for NGF signaling studies [71]. K252a, an efficient serine/threonine protein kinase inhibitor, can selectively block the effects of TrkA but not P75. LM11A-31 is a non-peptide small molecule ligand of the P75 neurotrophin receptor, which mimics the loop 1 of nerve growth factor and can completely bind to P75 but not TrkA [72, 73]. We found the decreased levels of β-catenin mRNA in the three cells induced by 100 ng/ml NGF can be up-regulated significantly by Ro 08-2750, K252a and LM11A-31. In CAOV3 and OVCAR3 cells, this elevation was observed at 24 hour time point after stimulation with more than 5 uM Ro 08-2750, 10 uM K252a , 5 nM LM11A-31 or by 100 uM K252a+5 nM LM11A-31 compared to only containing 100 ng/ml NGF group. In SKOV3 cells, the reduction in β-catenin mRNA induced by 100 ng/ml NGF can be blocked and result in an increase by more than 5 uM Ro 08-2750, 10 uM K252a or by 5 nM LM11A-31 and by 100 uM K252a+5 nM LM11A-31, but further down-regulated of β-catenin mRNA was present in 10 nM, 30 nM and 100 nM LM11A-31 groups (Figure 4A, 4C, 4E). Maybe an excess of LM11A-31 itself might inhibit the expression of β-catenin. The changes of cell protein expression were in relation to the mRNA expression in the three ovarian cancer cell lines (Figure 4B, 4D, 4F; Supplementary Figure S3A–3C).

**Figure 5:**
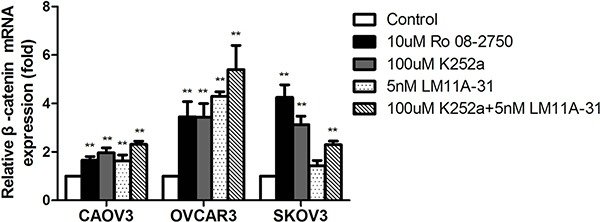
The down-regulation of β-catenin mRNA expression following NGF autocrine stimulation in ovarian cancer cells At 24 hour time point, the inhibitors of NGF/NGFRs can block the effect of endogenous NGF on β-catenin mRNA expression in serum-free culture conditions. The mRNA value represents fold difference compared to the control (serum-free medium group, **p < 0.01). Representative of three independent experiments.

Given that CAOV3, OVCAR3 and SKOV3 cells released endogenous NGF, we explored the changes of basal expression levels of β-catenin after treatment with NGF/NGFRs-related inhibitors without exogenous NGF to better verify whether the endogenous NGF was biologically active in regulating the expression of β-catenin in ovarian cancer cells. We chose 10 uM Ro 08-2750, 100 uM K252a, 5 nM LM11A-31 and 100 uM K252a+5 nM LM11A-31 which can completely block 100 ng/ml exogenous NGF-reduced expression of β-catenin to treat ovarian cancer cells that grown in serum-free medium containing 0.1% BSA. At 24 hour time point, qPCR analysis showed that a 1.63-to 2.3 fold increase of β-catenin mRNA in CAOV3 cells, 3.43-to 5.4 fold increase in OVCAR3 cells, 1.43-to 4.25 fold increase in SKOV3 cells compared to the control groups (serum-free medium group containing 0.1% BSA) after treatment with these inhibitors (Figure 5). Combination with the front experiments, we found the up-regulated folds under basal conditions were higher than the groups stimulated with exogenous NGF respectively. All of these results confirmed that NGF/NGFRs-related inhibitors can both block the effects of endogenous and exogenous NGF on β-catenin expression in ovarian cancer cells. It also reminded that the high affinity receptor TrkA and low affinity receptor P75 are both involved in the regulation of β-catenin.

### Effect of NGF and NGF/NGFRs-related inhibitors on β-catenin phosphorylation in ovarian cancer cells

**Figure 6:**
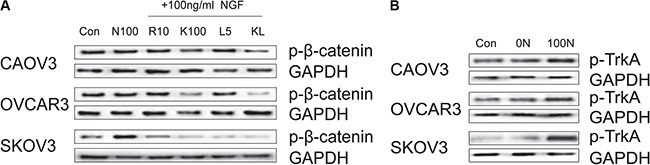
Effect of NGF and inhibitors of NGF/NGFRs on β-catenin phosphorylation and TrkA phosphorylation in ovarian cancer cells (**A**) The effect of 24 hour of treatment with NGF and the inhibitors of NGF/NGFRs on β-catenin phosphorylation in ovarian cancer cells (Con: non-stimulated, untreated ovarian cancer cells; N100: 100 ng/ml NGF; R10: 10 uM Ro 08-2750; K100: 100 uM K252a; L5: 5 nM LM11A-31; KL: 100 uM K252a+5 nM LM11A-31). (**B**) The TrkA phosphorylation in ovarian cancer cells under different culture conditions (Con: conventional culture condition; 0N: serum-free medium; 100N: 100 ng/ml NGF). GAPDH expression served as control. All of the figures are representative of three independent replicate studies.

To study the possible mechanism of β-catenin down-regulation or activation status in NGF-stimulated ovarian cancer cells, we assessed the effect of NGF and the inhibitors of NGF/NGFRs upon phosphorylation of β-catenin. Ovarian cancer cells were cultured for 24 hour by serum deprivation, and then were incubated for 24 hour with 100 ng/ml NGF, 10 uM Ro 08-2750+100 ng/ml NGF, 100 uM K252a+100 ng/ml NGF, 5 nM LM11A-31+ 100 ng/ml NGF, and 100 uM K252a+5 nM LM11A-31+100 ng/ml NGF. No visible effect on β-catenin phosphorylation was observed after 24 hour of treatment with 100 ng/ml NGF in CAOV3 and OVCAR3 cells, but in SKOV3 cells, there was a significant increase of β-catenin phosphorylation. The phosphorylation level of β-catenin was not changed in CAOV3 and OVCAR3 cells after treatment with 10 uM Ro 08-2750. On the other hand, 10 uM Ro 08-2750 decreased β-catenin phosphorylation induced by 100 ng/ml NGF in SKOV3 cells. The phosphorylation of β-catenin was inhibited by 100 uM K252a or 100 uM K252a+5 nM LM11A-31 in the three cells. There was no evident differences of β-catenin phosphorylation after treatment with 5 nM LM11A-31 compared to 100 ng/ml NGF group in CAOV3 and OVCAR3 cells, yet it can inhibit β-catenin phosphorylation by NGF-induced in SKOV3 cells (Figure 6A; Supplementary Figure S4A). We speculated that the β-catenin was activated by endogenous NGF when cells were cultured in serum deprivation. And exogenous NGF can further enhance phosphorylation of β-catenin in SKOV3 cells, but no significant effect on β-catenin phosphorylation in CAOV3 and OVCAR3 cells, which may be related to the higher endogenous NGF in them.

As we all know, TrkA is a high-affinity receptor for NGF, and it is widely linked to tumorigenesis. Increased phosphorylated TrkA (p-TrkA) expression has been described in advanced ovarian carcinoma, which may be useful as a prognostic and progression marker [74, 75]. The above-mentioned results suggest that inhibition of TrkA with K252a resulted in significant inhibition of β-catenin phosphorylation. Next, we examined the phosphorylation level of TrkA in ovarian cancer cells under different culture conditions to investigate the biological relevance of NGF and β-catenin. We found there were no remarkable difference about the phosphorylation level of TrkA in the three cells between the conventional culture condition and serum-free culture, whereas the level of phosphorylation of TrkA was significantly improved after 24 hour of treatment with 100 ng/ml NGF (Figure 6B; Supplementary Figure S4B). Our results showed that endogenous NGF can induce TrkA autophosphorylation, and exogenous NGF can further enhance the phosphorylation level of TrkA. The expression level and degree of activation of TrkA may play a major role in regulating the biological function of β-catenin. NGF can not only down-regulate the expression of β-catenin, but also affect the active state of β-catenin.

### NGF and inhibitors of NGF/NGFRs regulate the expression levels of BCL9-2 in ovarian cancer cells

**Figure 7:**
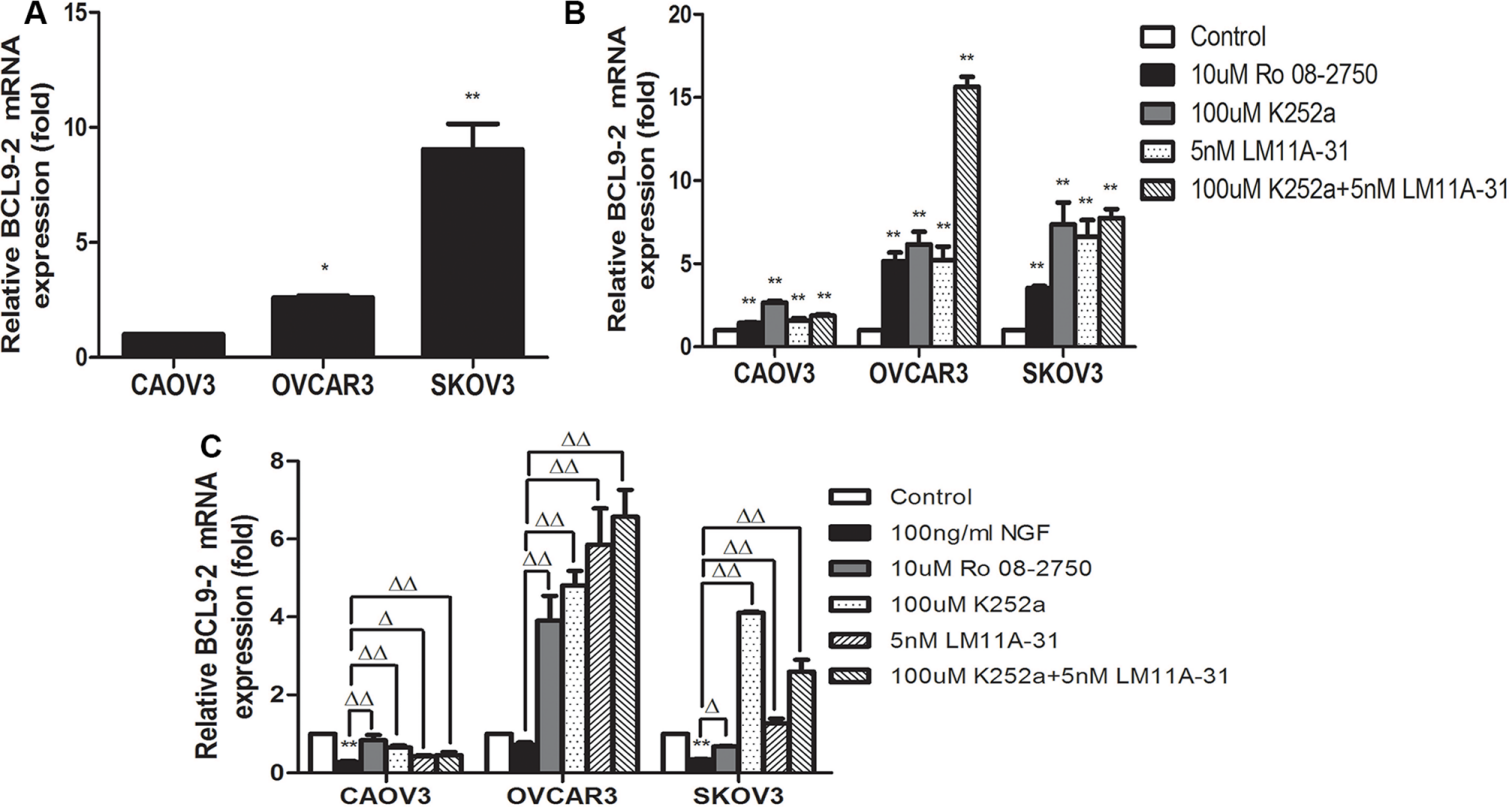
The effect of NGF or NGF/NGFRs-related inhibitors on BCL9-2 mRNA expression in ovarian cancer cells at 24 hour time point (**A**) The mRNA expression of BCL9-2 was observed in ovarian cancer cells under the conventional culture condition. The relative mRNA expression was normalized against GAPDH mRNA. The mRNA value represents fold difference compared to CAOV3 group under the conventional culture condition (*p < 0.05, **p < 0.01). (**B**) At 24 hour time point, the inhibitors of NGF/NGFRs can increase the mRNA expression of BCL9-2 by blocking the effect of endogenous NGF in serum-free culture conditions. The mRNA value represents fold difference compared to the control (serum-free medium group, **p < 0.01). (**C**) NGF decreased the mRNA levels of BCL9-2 in CAOV3, OVCAR3 and SKOV3 cells, and the inhibitors of NGF/NGFRs can block the effect of 100 ng/ml exogenous NGF on BCL9-2 mRNA expression in serum-free culture conditions. The mRNA value represents fold difference compared to the control (0 ng/ml NGF group, **p < 0.01) or 100 ng/ml NGF group (Δ*p* < 0.05, ΔΔ*p* < 0.01). Representative of three independent experiments.

The earlier studies demonstrated that β-catenin is an important pivot between cell adhesion and WNT signaling, which BCL9-2 is a control switch that flips the β-catenin pivot from cell adhesion to WNT signaling during normal and malignant development. The overexpressed BCL9-2 can promote migratory activity and scattering, which induces changes in epithelial cells that are akin to an epithelial-mesenchymal transition and involves in tumorigenesis or development in the epithelial tissues [76–78]. Here, we found that BCL9-2 mRNA was expressed in CAOV3, OVCAR3 and SKOV3 cells (Figure 7A). Combined with previous experimental results, we speculated that the simultaneous deregulation of β-catenin and BCL9-2 in the same ovarian cancer cell may have therefore synergistic effect leading to malignant progression of ovarian cancer. In order to ascertain the physiological mechanism of NGF how to regulate the expression and activity of β-catenin, we measured the mRNA expression changes of BCL9-2 after treatment with 10 uM Ro 08-2750, 100 uM K252a, 5 nM LM11A-31 and 100 uM K252a+5 nM LM11A-31 under basal culture condition. Interestingly, the inhibitors of NGF/NGFRs increased the mRNA expression intensity of BCL9-2 significantly, which implied that endogenous NGF may decrease the levels of BCL9-2 (Figure 7B). We next analyzed the mRNA expression level of BCL9-2 following the stimulation with 100 ng/ml NGF, 10 uM Ro 08-2750+100 ng/ml NGF, 100 uM K252a+100 ng/ml NGF, 5 nM LM11A-31+100 ng/ml NGF and 100 uM K252a+5 nM LM11A-31+100 ng/ml NGF at 24 hour time point. The results indicated that NGF reduced the levels of BCL9-2 and the changes showed a similar tendency to the expression of β-catenin induced by NGF, which can be blocked by the inhibitors of NGF/NGFRs (Figure 7C). Taken together, these findings demonstrated that NGF may affect the expression or activity of β-catenin by regulating the expression levels of BCL9-2 in ovarian cancer cells.

### NGF and related inhibitors of NGF/NGFRs modulate the expression levels of WNT/β-catenin downstream target genes in ovarian cancer cells

**Figure 8:**
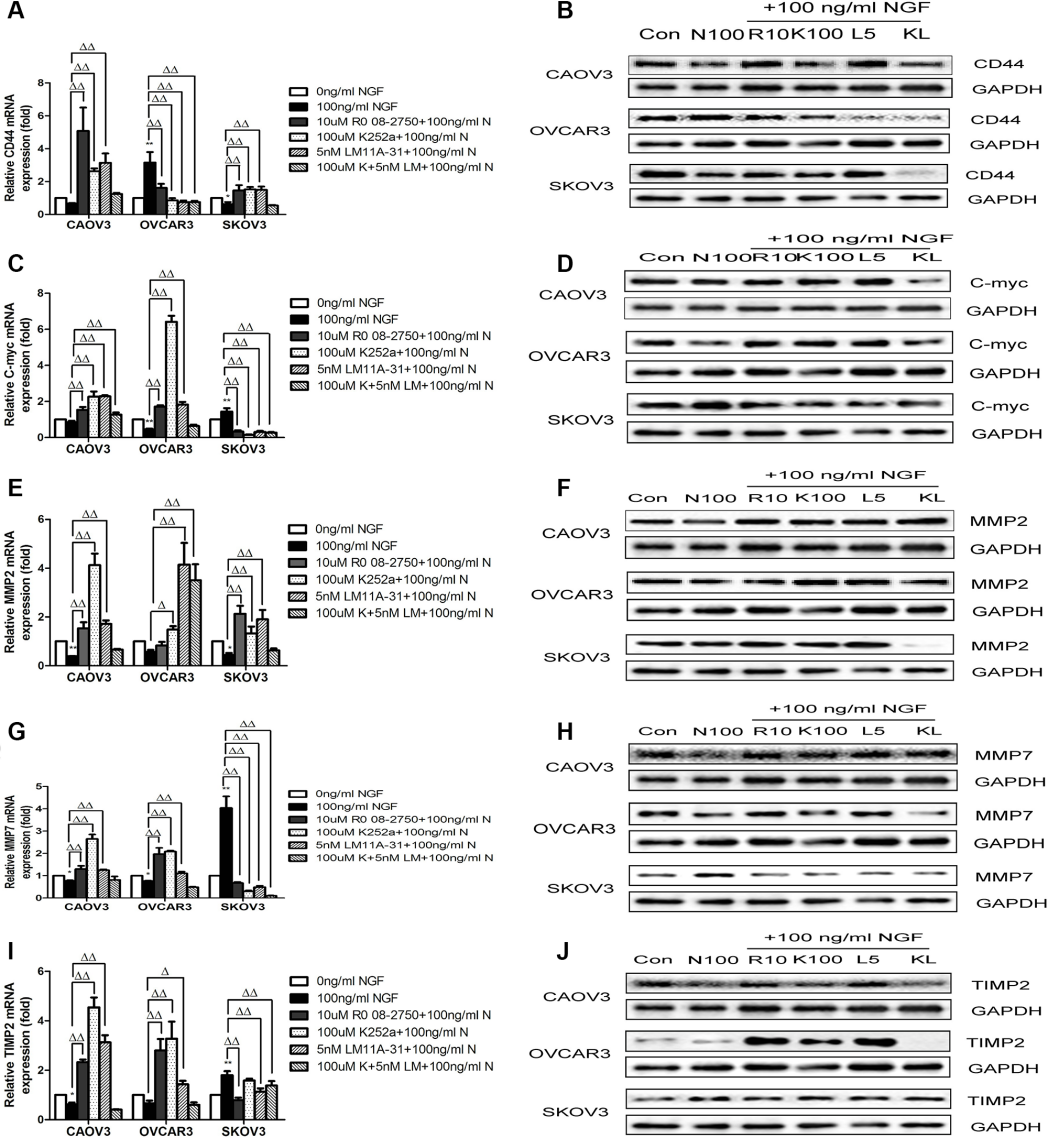
NGF and related inhibitors of NGF/NGFRs modulate the expression levels of WNT/β-catenin downstream target genes on ovarian cancer cells (**A** and **B**) The NGF and NGF/NGFRs-related inhibitors modulate the mRNA and protein expression levels of CD44 in ovarian cancer cells at 24 hour time point. (**C** and **D**) The NGF and NGF/NGFRs-related inhibitors modulate the mRNA and protein expression levels of C-myc in ovarian cancer cells at 24 hour time point. (**E** and **F**) The NGF and NGF/NGFRs-related inhibitor modulate the mRNA and protein expression levels of MMP2 in ovarian cancer cells at 24 hour time point. (**G** and **H**) The NGF and NGF/NGFRs-related inhibitor modulate the mRNA and protein expression levels of MMP7 in ovarian cancer cells at 24 hour time point. (**I** and **J**) The NGF and NGF/NGFRs-related inhibitor modulate the mRNA and protein expression levels of TIMP2 in ovarian cancer cells at 24 hour time point. (100 ng/ml N: 100 ng/ml NGF; Con: 0 ng/ml NGF; N 100:100 ng/ml NGF; R10:10 uM Ro 08-2750+100 ng/ml NGF; K100:100 uM K252a+100 ng/ml NGF; L5:5 nM LM11A-31+100 ng/ml NGF; KL: 100 uM K252a+5 nM LM11A-31+100 ng/ml NGF). mRNA value represent fold difference in relation to the untreated control (0 ng/ml NGF, *p < 0.05, **p < 0.01.) and 100 ng/ml NGF group (Δ*p* < 0.05, ΔΔ*p* < 0.01.) Data represent three replicate studies and mean ± sd. GAPDH ex-pression served as control in western blot assay. Representative of three independent experiments.

To further identify the regulation effects of NGF/NGFRs on WNT/β-catenin pathways in ovarian cancer cells, we examined the expression changes of WNT/β-catenin downstream target genes that are related to tumor proliferation, adhesion, invasion and metastasis, such as CD44, C-myc, MMP2, MMP7 and TIMP2, following the stimulation with 100 ng/ml NGF, 10 uM Ro 08-2750, 100 uM K252a, 5 nM LM11A-31 and 100 uM K252a+5 nM LM11A-31, and sustained for 24 hour period of observation. We observed the expression of CD44 was decreased 1.48-fold in CAOV3 cells and 1.59-fold in SKOV3 cells with the stimulation of 100 ng/ml NGF, but the difference was not significant compared to 0 ng/ml NGF group in CAOV3 cells. However, 100 ng/ml NGF can significantly increase the levels of CD44 (3.15-fold) in OVCAR3 cells. The increase or decrease of CD44 expression induced by 100 ng/ml NGF can be reverse regulation by their inhibitors. We also found the levels of CD44 was no elevation by simultaneously blocking TrkA and P75 receptors in CAOV3 and SKOV3 cells (Figure 8A, 8B; Supplementary Figure S5A). The overexpression of transcription-regulatory oncoprotein C-myc has been reported in most types of human malignancies, including ovarian cancers, which controls genes involved in cell cycle progression, cell growth and apoptosis [79–82]. Here, we found the levels of C-myc was down-regulated 1.19-fold in CAOV3 cells and 2.22-fold in OVCAR3 cells, but up-regulated 1.45-fold in SKOV3 cells after treatment with 100 ng/ml NGF. The effects of NGF can be blockade by antagonists of NGF/NGFRs (Figure 8C, 8D; Supplementary Figure S5B). In CAOV3 and OVCAR3 cells, MMP2 decreased 2.56-fold *vs* 1.75-fold, MMP7 reduced 1.28-fold *vs* 1.32-fold, and TIMP2 declined 1.61-fold *vs* 1.52-fold respectively following the stimulation with 100 ng/ml NGF. And in SKOV3 cells, MMP2 decreased 2.27-fold, nevertheless, MMP7 increased 4.04-fold and TIMP2 increased 1.8-fold stimulated with 100 ng/ml NGF. The antagonists of NGF/NGFRs can abolish the effects of NGF (Figure 8E–8J; Supplementary Figure S5C–5E). These results suggest that the increase or decrease of gene transcription of C-myc, CD44, MMP2, MMP7 and TIMP2 might be a result of activation about metastasis related transcriptional program in ovarian cancer cells by NGF.

### NGF/NGFRs modulate the migration of ovarian cancer cells

**Figure 9:**
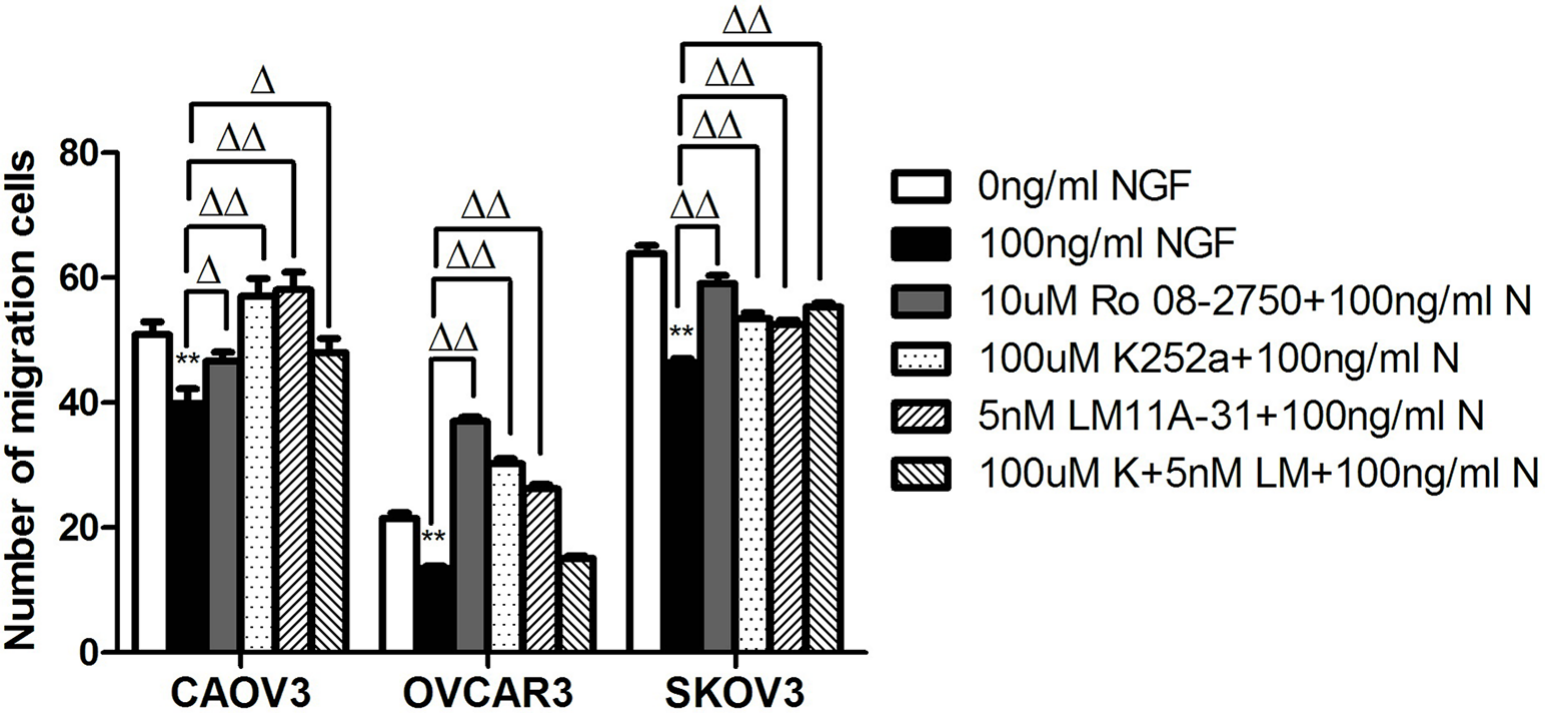
The stimulation of ovarian cancer cell lines with NGF and NGF/NGFRs-related inhibitors changed the migration ability of ovarian cancer cells by transwell assay The cells migrated PET membrane were reduced after treatment with NGF, but the inhibitors of NGF/NGFRs can block this effect. (**p < 0.01, *vs*. 0 ng/ml NGF; Δ*p* < 0.05, ΔΔ*p* < 0.01, *vs*.100 ng/ml NGF) Representative of three independent experiments.

To determine whether the NGF/NGFRs can regulate the migration of ovarian cancer cells through WNT/β-catenin signaling pathway, transwell migration assays (two dimensional growth environment, 2D) and microfluidic chip experiments (3D growth condition) were done. After pretreatment with NGF and related inhibitors of NGF/NGFRs in serum-free medium containing 0.1% BSA for 1 hour, the cells suspension of CAOV3, OVCAR3 and SKOV3 were added to the upper chamber of the transwell, and conventional medium supplemented 10% FBS were added to the lower chamber of the transwell to induce cell migration. The number of migration cells of CAOV3, OVCAR3 and SKOV3 had decreased 1.28-fold, 1.59-fold and 1.37-fold respectively after stimulation with 100 ng/ml NGF at 24 hour time point. And it can be blocked by 10 uM Ro 08-2750, 100 uM K252a, 5 nM LM11A-31 or the combined effects of 100 uM K252a and 5 nM LM11A-31, but in OVCAR3 cells, the blockade was not obvious by 100 uM K252a+5 nM LM11A-31(Figure 9).

**Figure 10:**
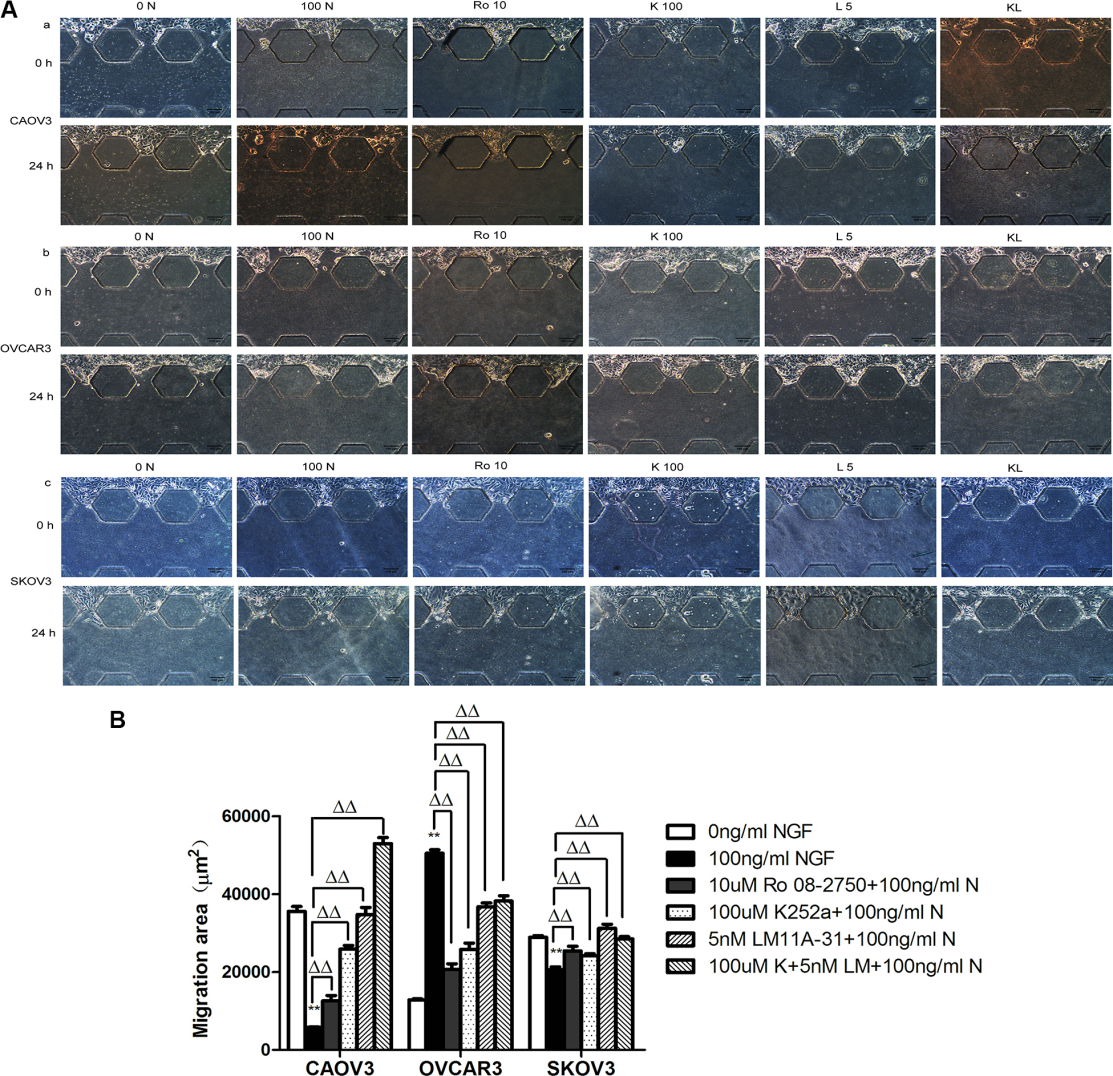
The changes of migration area migrated into the 3D matrigel of ovarian cancer cells following the stimulation of the NGF and related inhibitors of NGF/NGFRs in microfluidic device The cell suspensions were injected to the upper channel, the matrigel were added into the middle channel and the serum-free medium containing 0.1%BSA were injected to the lower channel. (**A**) Migration of ovarian cancer cells in microfluidic channels stimulated with NGF and related inhibitors of NGF/NGFRs at 24 hour time point. The scale bar is 100 μm and the results are one representative of three replicate studies. (Note: 0 N: 0 ng/ml NGF; 100 N: 100 ng/ml NGF; R10:10 uM Ro 08-2750+100 ng/ml NGF; K100:100 uM K252a+100 ng/ml NGF; L5:5 nM LM11A-31+100 ng/ml NGF; KL: 100 uM K252a+5 nM LM11A-31+100 ng/ml NGF.) (**B**) Statistical analysis of ovarian cancer cell migration area treated with NGF and NGF/NGFRs-related inhibitors. (**p < 0.01, *vs*. 0 ng/ml NGF; ΔΔ*p* < 0.01, *vs*.100 ng/ml NGF).

**Figure 11:**
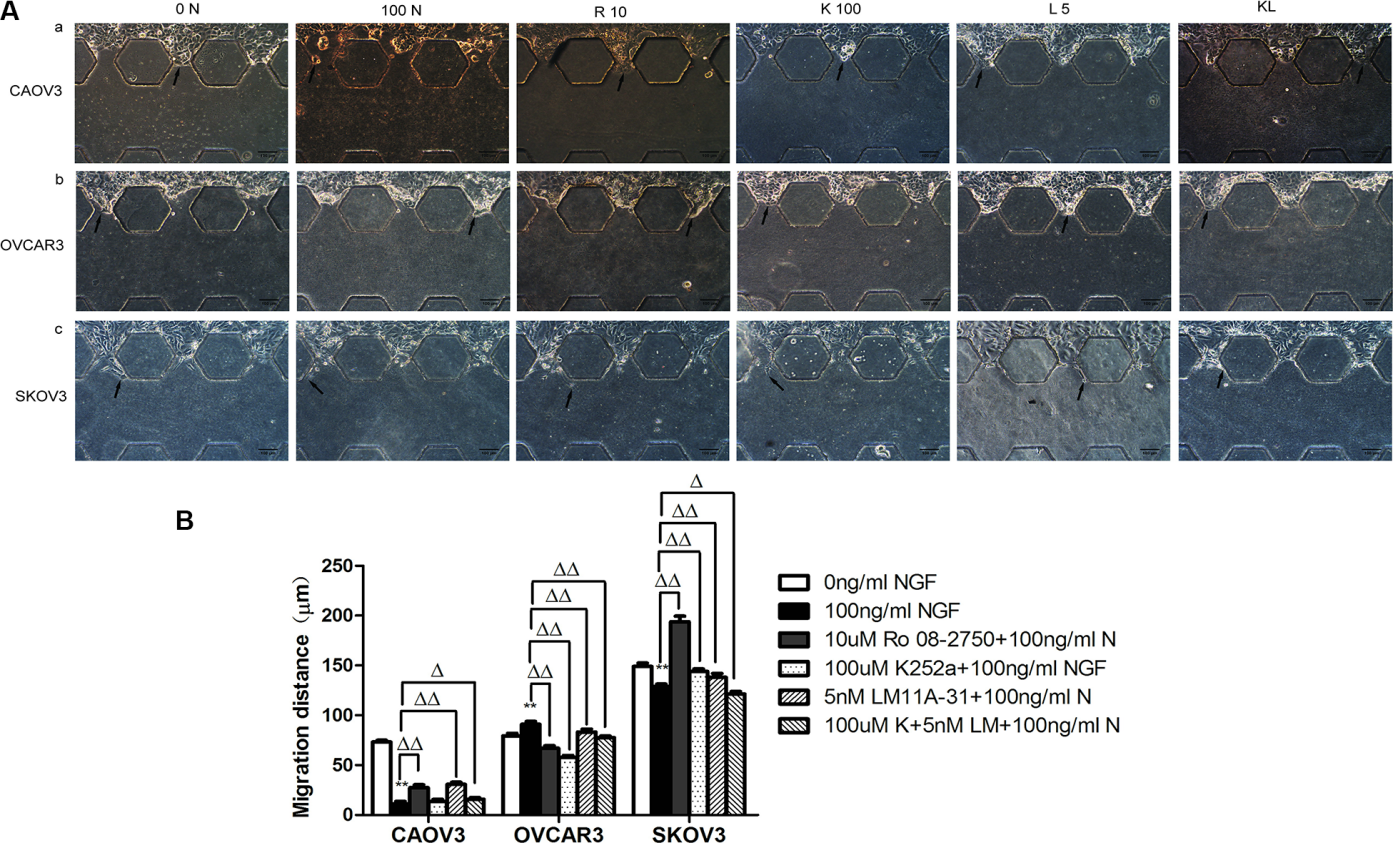
The maximum migration distance of ovarian cancer cells migrated into matrigel following the stimulation of the NGF and related inhibitors of NGF/NGFRs in microfluidic device at 24 hour time point (**A**) Migration of ovarian cancer cells in microfluidic channels stimulated with NGF and related inhibitors of NGF/NGFRs. The scale bar is 100 μm and the results are one representative of three replicate studies. (**B**) Statistical analysis of ovarian cancer cell maximum migration distance treated with NGF and NGF/NGFRs-related inhibitors. (**p < 0.01, *vs*. 0 ng/ml NGF; Δ*p* < 0.05, ΔΔ*p* < 0.01, *vs*.100 ng/ml NGF.)

We used microfluidic device to construct 3D model to mimic and monitor the growth condition of ovarian cancer cells *in vitro.* The main advantage of microfluidic systems is that using small quantities of cells and reagents to constitute a precise control of spatial and temporal environments and to visualize the cellular events in real time [83, 84]. After stimulation with 100 ng/ml NGF for 24 hours, in CAOV3 cells, the migration area and maximum distance into the 3D matrigel decreased 6.25-fold and 6.67-fold respectively; In SKOV3 cells, the migration area and maximum distance reduced 1.41-fold and 1.15-fold respectively; But in OVCAR3 cells, a 3.92-fold and 1.14-fold increase of the migration area and maximum distance respectively was observed after 24 hour of treatment with 100 ng/ml NGF. All of these biological effects can be antagonized by 10 uM Ro 08-2750, 100 uM K252a, 5 nM LM11A-31 or the combined effects of 100 uM K252a and 5 nM LM11A-31(Figures 10, 11).

**Figure 12:**
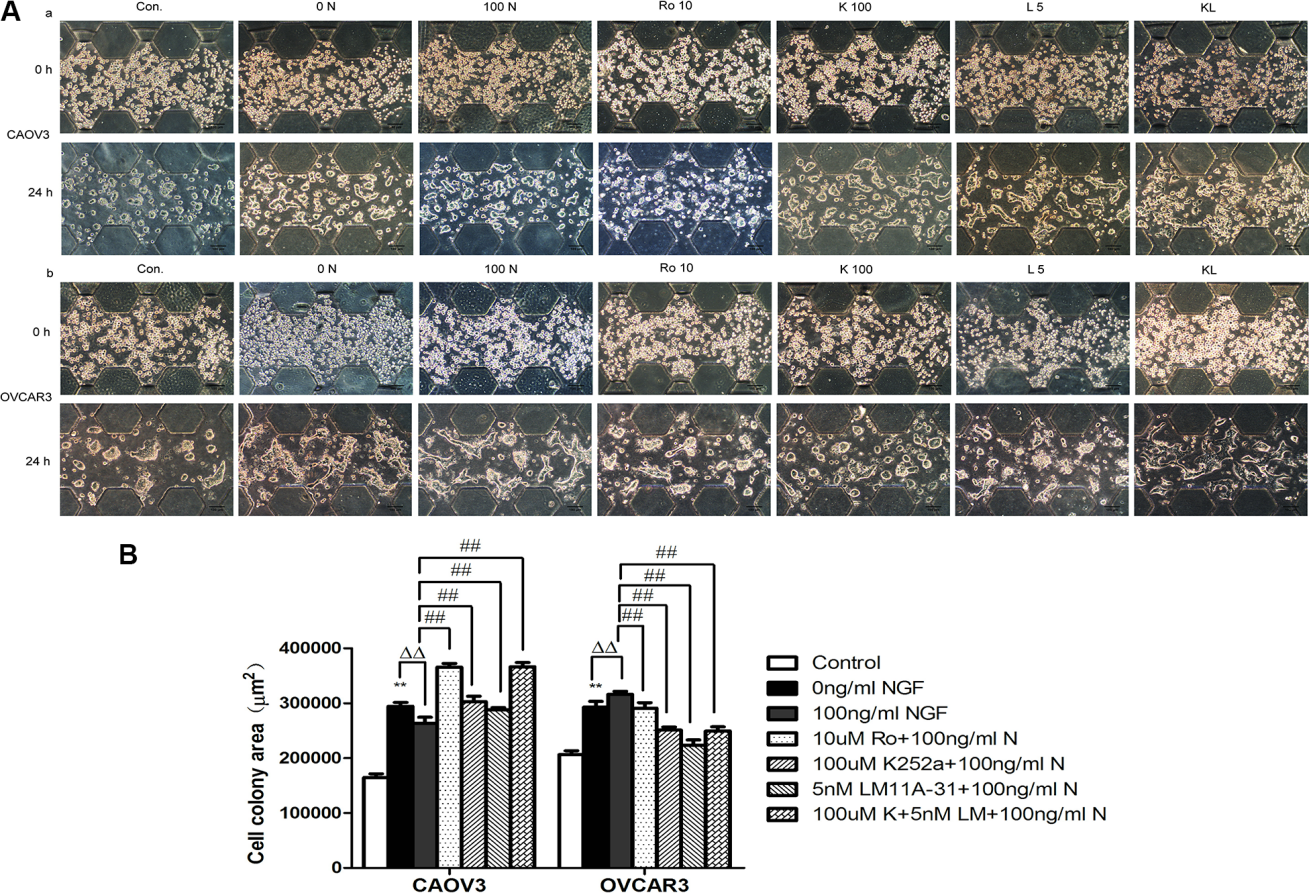
Ovarian cancer cell colony formation with the stimulation of the NGF and related inhibitors of NGF/NGFRs in microfluidic device at 24 hour time point (**A**) The mixture of ovarian cancer cells and matrigel (2:1, v:v) were injected to the middle channel. After the cell-gel suspensions polymerization, the NGF and related inhibitors of NGF/NGFRs diluted with the serum-free medium containing 0.1%BSA were injected into both sides of the channel. The scale bar is 100 μm. (**B**) Statistical analysis of ovarian cancer cell colony formation area with the stimulation of the NGF and related inhibitors of NGF/NGFRs in microfluidic device at 24 hour time point. (**p < 0.01, *vs*. Control: the medium containing 10% serum; ΔΔ*p* < 0.01, *vs*. 0 ng/ml NGF; ^##^*p* < 0.01, *vs*. 100 ng/ml NGF). The results are representative of three experiments and the values are expressed as mean ± sd.

We observed that CAOV3 and OVCAR3 cells tended to form cell colony in the process of growth and spread in a pattern of collective cell migration, especially in the case of unfavorable culture conditions. However, the “lead-cells” of SKOV3 extended filopodial projections to the surroundings in the growth process. And so, we chose CAOV3 and OVCAR3 cells to ascertain the functional role of NGF/NGFRs on ovarian tumor collective migration in a relatively real 3D growth environment. The cell-matrigel mixture were seeded into the middle channel of the microfluidic device and fully polymerized. After the mixture of cell-gel polymerized completely, culture medium, NGF and related inhibitors of NGF/NGFRs were injected to the both sides of the channel to culture for 24 hours. As shown in Figure 12, 3D encapsulated CAOV3 and OVCAR3 formed many cell colony. The single cell colony area more than 600 μm^2^ were collected and summarized. The results showed that the colony area of CAOV3 and OVCAR3 increased 1.79-fold and 1.42-fold of 0 ng/ml NGF group (serum-free medium containing 0.1% BSA) compared to normal culture conditions (Control, containing 10% serum medium). It suggested that CAOV3 and OVCAR3 cells were easier to form cell colony under the sub-conditions compared to normal culture conditions. The colony area of 100 ng/ml NGF group in CAOV3 down-regulated 1.12-fold compared to 0 ng/ml NGF group, but augmented 1.08-fold in OVCAR3 cells. The inhibitors of NGF/NGFRs can partially or completely block these effects. Together, these results indicated that NGF/NGFRs can regulate the migration behavior of ovarian cancer cells, relating to cell types and growth conditions.

## DISCUSSION

It is well known that ovarian carcinoma is easy to migrate and transform to the advanced-stage, which is rarely cured and long-term survival is facing serious challenges. The comprehension to control mechanisms of clinical behavior is essential to establish molecular therapeutic strategies for ovarian cancer patients. Both NGF/NGFRs and WNT/β-catenin signaling pathways are critical for the initiation, development, progression and prognosis of the patients with ovarian cancer [85, 86]. NGF is an essential neurotrophin and a key modulator of neuro-endocrine immune axis that serves diverse biological functions [87–89]. The two biological receptors for NGF, TrkA and P75, collaborate to generate high-affinity binding sites for NGF [44, 90]. NGF binding to TrkA receptor activates TrkA autophosphorylation, which then recruits various intracellular adaptors to in turn lead to the activation of a variety of signaling networks [91, 92]. TrkA signaling can be modulated by P75 that is the first described co-receptor of TrkA, which increases both the sensitivity and specificity of TrkA to NGF, and delays TrkA ubiquitination and sustaining its phosphorylation [93–95]. In addition, P75 plays many diverse roles in cell survival, death, migration, and so forth through the activation of nuclear factor NF-κB, Jun kinase (JNK) and caspases [96–100]. This fully proves that the effect of P75 on the cellular response to neurotrophins is complex, and may depend on the concentration of ligand, the ratio of receptors, the cell type and stage of differentiation in which it is expressed [101, 102]. In a variety of human cancers, NGF has been shown to play a key role in tumor cell growth, proliferation, invasion, angiogenesis, and so on [103–106]. In our cell models, NGF and NGFRs were found to be expressed in ovarian cancer cells simultaneously. Endogenous NGF induced phosphorylation of TrkA in conventional or conditioned culture medium, and 100 ng/ml NGF can obviously enhance the effect. NGF is able to stimulate the cellular proliferation of human ovarian cancer cells, participates in pathological angiogenic processes of ovarian cancer directly or indirectly that may contribute to the growth, aggressiveness and low survival rates for ovarian cancer patients [107, 108]. We also found NGF/NGFRs and β-catenin co-expression were observed in CAOV3, OVCAR3 and SKOV3 cells. Up to now, however, there is little literature to examine their interaction and the resulting intracellular signaling between NGF/NGFRs and β-catenin in human cancers. The β-catenin protein is a multifunctional oncogenic protein and a vital component of the canonical WNT/β-catenin signaling pathway in many human cancers [109]. It can be activated and modulated by WNT ligands, a protein family consists of 19 ligands that plays different roles in signal transduction, through canonical and non-canonical WNT pathways controlling ovarian carcinogenesis [110–112]. *In vitro* studies revealed that the silence of β-catenin can inhibit the proliferation and decrease capability of colony formation in A2780 ovarian cancer cells, a similar effect was observed in colorectal cancer, lung cancer and glioblastoma cells [113–116]. It means that β-catenin might be required for proliferation and migration of some cancer cells, although there are few studies to illuminate the precise mechanisms by which cancer cells acquire or regulate such expression. Especially worthy of attention is that we demonstrated for the first time that NGF is associated with β-catenin in human ovarian cancer migration in this article. Our results showed that NGF can down-regulate the expression intensity of β-catenin in ovarian cancer cells, but the regulation mechanism were not known. It was previously reported that BCL9-2, a member of novel BCL9/legless oncogene family, was identified as essential co-activator and modulator of WNT/β-catenin signals dependent regulation of target gene transcription in development and tumorigenesis. And it was a molecular switch between the adhesive and transcriptional functions of β-catenin [117–122]. In this report, we showed that the mRNA expression of BCL9-2 was reduced after treatment with NGF. Moreover, the expression changes showed a similar tendency to the expression of β-catenin induced by NGF, which can be blocked by the inhibitors of NGF/NGFRs. It revealed that NGF may affect the expression and function of β-catenin by regulating the expression level of control switch BCL9-2.

The metastatic capability of cancer cells is considered to be an important factor for cancer death [123]. And the breakage or degradation of extracellular matrix (ECM) and basement membrane (BM) is a key step in tumor progression, where the specific adhesion molecules and MMPs are key dynamical factors in the interaction between tumor cells and microenvironment [124–128]. Ovarian cancer is a highly metastatic malignant tumor that adhesion and invasion are two critical processes for ovarian tumor dissemination and metastasis. Several downstream target genes of WNT/β-catenin signaling, such as CD44, C-myc, MMP2, MMP7 and TIMP2, can affect tumor growth, proliferation, invasion and metastasis. CD44 proteoglycan is a multifunctional membrane receptor that expressed in a wide variety of cell types, including tumor types and cancer stem cells, and involves in cell adhesion, motility, metastases and so on [129–131]. C-myc is an oncogene and activated in many types of human malignancy, which is identified to be the first target oncogene in WNT signaling pathway. The activated C-myc transcription factor may participate in the regulation of numerous processes, such as epithelial-mesenchymal transition, cell proliferation, cycle progression, angiogenesis, metastasis [132, 133]. MMPs and tissue inhibitors of metalloproteinases (TIMPs) play crucial regulatory roles in the homeostasis of the extracellular matrix, which are markers of ovarian cancer cell proliferation and invasiveness. MMP2 is a member of MMPs family involved tissue remodeling, degradation of collagens and interaction with other extracellular matrix macromolecules, which linked to enhanced tumor invasion/metastasis in several *in vitro* and *in vivo* model systems [134, 135]. MMP7, a soluble metalloproteinase and an established WNT/β-catenin target gene, has been repeatedly linked to tumor progression and metastasis in various types of cancers [136–138]. TIMP2 is an important endogenous inhibitor of MMPs which combines with MMP2 preferentially and can regulate the tumor growth, invasion and angiogenesis by a variety of mechanisms for MMP-dependent or MMP-independent pathways [139–142]. All of these factors are considered to be very important for metastasis of tumor cells. In our work, we found NGF decreased the expression of CD44 in CAOV3 and SKOV3 cells, but increased in OVCAR3 cell. It is possible that NGF serves as a bridge between TrkA and CD44, which the TrkA/CD44 complex upon NGF stimulation leads to the activation of CD44 pathway [143]. Meanwhile, we found the inhibitor of p75 can block the effect of NGF, which meant that P75 receptor involved in the regulation of CD44, too. CD44 binding with its major ligand Hyaluronic acid, a major component of the peritoneum which is most important site for ovarian cancer metastases, interacts with the tumor stroma and the tumor microenvironment as relevant to ovarian cancer metastatic growth [144–146]. C-myc is an important clinicopathological parameter of ovarian tumors such as degree of malignancy, histological type and prognosis [147–149]. Our results showed the expression of C-myc was decreased in CAOV3 and OVCAR3 cells, but increased in SKOV3 cells stimulated by NGF. Some studies have been published on the prognostic value of MMP2, MMP7 and TIMP2 in ovarian cancers, but it is still controversial. Most of them overexpressed in tumors, peritoneal implants or metastatic lesions that associated with poor outcome, but others predicted better survival [150–154]. Anyway, the effect of MMPs on the degradation of extracellular matrix to promote cell movement and migration is no doubt. And here, we detected that NGF/NGFRs decreased MMP2 expression level of the three cells, and it was consistent with previous research views [155, 156]. MMP7 promotes invasion and migration of tumor cells via extracellular cleavage of E-cadherin, and also can activate other MMPs, such as proMMP2 and proMMP9, to facilitate ovarian tumor invasion [157–159]. We observed that the expression level was correspondingly changed by NGF and NGF/NGFRs-related inhibitors. TIMP2 was strong in ovarian carcinomas, however, the function of TIMP2 in carcinogenesis of the ovary are inconsistent and seem to depend on the detection methods and the histological type of tumors [160–162]. Results from ours demonstrated that NGF/NGFRs decreased the expression of TIMP2 in CAOV3 and OVCAR3 cells, but increased the expression in SKOV3 cell. The imbalance between MMPs and TIMPs is an important factor to cause the degradation of extracellular matrix and promote the invasion and migration of ovarian cancer cells. We finally described the movement and migration behavior of ovarian cancer cells involving stimulation of NGF. Particularly, we have successfully applied the 3D microfluidic technology, which can precisely control the spatial and temporal environment and visualize the cellular events in real time to monitor the changes of cell behavior following the stimulation with NGF or the inhibitors of NGF/NGFRs. The migration area and maximum migration distance of CAOV3 and SKOV3 were downregulated with the stimulation of 100 ng/ml NGF, but upregulated in OVCAR3. The variation trend of cell colony area induced by NGF between CAOV3 and OVCAR3 was not consistent with each other, too. We have also seen that the changes of number of migration cells stimulated with NGF and the inhibitors of NGF/NGFRs in transwell assay were different from those in 3D microenvironment. However, these results highlight the view that the occurrence and development of ovarian carcinoma is a result of multiple factors, which we need to consider multiple distinct clinicopathological implications in ovarian carcinogenesis and progression.

Although more work is needed to fully elaborate the mechanism of ovarian cancer cell migration mediated by NGF through WNT/β-catenin pathway, the results presented here may provide some new insights in the biological activities of NGF/NGFRs and WNT/β-catenin signaling in ovarian cancer growth and spread. And therefore, there lies some new therapeutic opportunities that anchoring to the interaction between NGF signaling and WNT/β-catenin pathway in ovarian cancer.

## MATERIALS AND METHODS

### Reagents and antibodies

Cell culture reagents were purchased from Life Technologies (Carlsbad, CA, USA). RNA extraction and Ultra ECL chemiluminescence system reagents were purchased from BioTeke (Beijing, China). PrimeScript™RT reagent Kit with gDNA Eraser was purchased from Takara Biotechnology (Takara Bio Inc, Shiga, Japan). SsoFast^™^EvaGreen^®^ Supermix was purchased from Bio-Rad Laboratories (California, USA). Recombinant Human β-NGF was purchased from Peprotech (Rocky Hill, NJ, USA). Anti-β-NGF, anti-TrkA, anti-P75 and anti-GAPDH antibodies were purchased from Epitomics (Burlingame, CA, USA). Ro 08-2750 was purchased from R&D Systems (Minneapolis, MN, USA). K252a was purchased from Biovision (Milpitas, CA, USA). LM11A-31 was purchased from Sigma-Aldrich (Saint Louis, MO, USA). Anti-β-catenin and anti-C-myc were purchased from Arigo Biolaboratories (Taiwan, China). Anti-CD44, anti-MMP2, anti-MMP7 and anti-TIMP2 were purchased from OriGene Technologies, Inc (Rockville MD, USA). p-CTNNB1-S552 and p-TrkA-Y490 antibodies were purchased from ABclonal Technology (Woburn, MA, USA). PVDF membranes were purchased from Millipore (Bedford, MA, USA).

### Ovarian cancer cell culture

Human ovarian cancer cell lines A2780, SKOV-3 , OVCAR-3 and CAOV3 were purchased from American Type Culture Collection (Rockville, MD,USA). A2780, SKOV-3 and OVCAR-3 were grown in a base culture media of DMEM and CAOV3 was incubated in RPMI-1640 medium, which all were supplemented with 10% fetal bovine serum and 100 units/ml penicillin/streptomycin. The four cell lines were maintained at 37°C in a humidified incubator with 5% CO_2_. In confluent state, cells were washed twice with PBS and once with serum-free, and then were cultured in a base culture media with 0.1% bovine serum albumin (BSA) for 24 hours in order to eliminate the influence of serum. Next, the cells in the conditioned medium were stimulated with different concentrations of NGF and other related inhibitors. At last, the treated cells were collected at a few chosen time points for further experimental detection.

### RNA extraction and quantitative real-time PCR analysis

Total RNA was extracted from the four cultured ovarian cancer cell lines with TRIpure reagent (BioTeke, Beijing, China) according to the instructions of the manufacturer. The complementary strand of DNA (cDNA) was synthesized by reverse transcription of 1 μg of total RNA employing PrimeScript™ RT reagent Kit with gDNA Eraser (TaKara Bio Inc, Japan). Quantitative real-time PCR reaction was performed with specific primers of the human genes to detect the expression levels of genes by SsoFast EvaGreen Supermix using iQ^™^ 5 real-time PCR thermal cycle instrument (Bio-Rad, California, USA). The threshold cycles (Ct) were determined and the quantitation of gene expressions were normalized to the housekeeping gene GAPDH (ΔCt). Experiments were done in triplicate independently. The sequences of primers were shown in Table 1. The primer sequences of GAPDH, NGF, C-myc, CD44, MMP2, MMP7, TIMP2 and BCL9-2 were from http://pga.mgh.harvard.edu/primerbank/; The primer sequences of P75 were from http://www.origene.com/qPCR/Primers.aspx; The primer sequences of β-catenin were from http://medgen.ugent.be/rtprimerdb/assay; The primer sequences of TrkA were designed using Primer-Blast from sequence submitted to the Genbank. The concentrations of all primers were 400 nmol/L.

**Table 1:** Primers sequences used for quantitative real-time PCR

Gene symbol	Forward/Reverse primers	Primer sequences 5′–3′
GAPDH	F	CTGGGCTACACTGAGCACC
	R	AAGTGGTCGTTGAGGGCAATG
NGF	F	GGCAGACCCGAACATTACT
	R	CACCACCGACCTCGAAGTC
TrkA	F	GAGGGCCTAGGAGCAGTAAG
	R	GAGATCTAGCAGCCCGCAAC
P75	F	CCTCATCCCTGTCTATTGCTCC
	R	GTTGGCTCCTTGCTTGTTCTGC
β-catenin	F	GCTGGGACCTTGCATAACCTT
	R	ATTTTCACCAGGGCAGGAATG
C-myc	F	AATAGAGCTGCTTCGCCTAGA
	R	GAGGTGGTTCATACTGAGCAAG
CD44	F	CTGCCGCTTTGCAGGTGTA
	R	CATTGTGGGCAAGGTGCTATT
MMP2	F	TACAGGATCATTGGCTACACACC
	R	GGTCACATCGCTCCAGACT
MMP7	F	GAGTGAGCTACAGTGGGAACA
	R	CTATGACGCGGGAGTTTAACAT
TIMP2	F	GCTGCGAGTGCAAGATCAC
	R	TGGTGCCCGTTGATGTTCTTC
BCL9-2	F	TGAACCTGAACGTGCAGATGA
	R	CCCTGGTTGGGAAACTGTG

### Western blot analysis

Ovarian cancer cells were seeded (5 × 10^6^ cells per dish) in circular culture dishes (60 × 15 mm per dish) and cultured with medium supplemented with 10% fetal bovine serum to 80% cell fusion. The cells were starved with serum-free medium containing 0.1%BSA, lasting for 24 hours. The cells were stimulated with or without different concentrations of NGF, Ro 08-2750, K252a, LM11A-31. Cell cultures (around 10^6^ cells) were homogenized for 30 min on ice in RIPA lysis buffer (Sigma, Saint Louis, USA) with proteinase inhibitors in the set of time points. After centrifugation for 5 min at 12,000 rpm, protein content was quantified using the BCA Protein Assay kit (Pierce, Rockford, USA). 10 μg of total protein was denatured and fractionated in an 8–10 % SDS-PAGE gel and then transferred onto apolyvinyldifluoride (PVDF) membrane. After blocking with 5% fat free milk, membranes were immunoblotted with anti-GAPDH, anti-NGF, anti-TrkA, anti-P75, anti-β-catenin, anti-C-myc, anti-MMP2, anti-MMP7, anti-CD44, anti-TIMP2, p-CTNNB1-S552 and p-TrkA-Y490 antibodies in TBST overnight at 4°C. After washing several times in TBST, bound antibody was visualized using peroxidase conjugated goat anti-mouse IgG or peroxidase conjugated goat anti-rabbit IgG (Origene, Rockville MD, USA) and the enhanced chemiluminescence (ECL) detection system (Amersham Pharmacia Biotech, Buckinghamshire, UK). Negative controls consisted of antibody in the absence of lysate. The band intensity of proteins from the immunoblots was quantified by densitometric scanning analysis using the Image Lab Program (Bio-rad, USA).

### Transwell assay

Falcon 24-well format transwell with a polyethylene terephthalate (PET) membrane/8 μm pores (BD Biosciences, USA) were used for ovarian cancer cells migration assays. Ovarian cancer cells (1 × 10^5^) pre-treated with 0 ng/ml NGF, 100 ng/ml NGF, 100 ng/ml NGF+10 uM Ro 08-2750, 100 ng/ml NGF+100 uM K252a, 100 ng/ml NGF+5 nM LM11A-31 and 100 ng/ml NGF+100 uM K252a+5 nM LM11A-31 for 1 hour were seeded into the upper chamber together with the treatments and medium supplemented with 10% FBS added to the lower chamber. Migration assays were incubated for 24 hours at 37°C and 5% CO_2_. Migrated cells on the lower surface were washed with PBS, and the cells on the top surface of the upper chamber were removed by wiping with a cotton swab. The cells that migrated to the bottom surface of the upper chamber were fixed with 4% paraformaldehyde and stained by 0.1% crystal violet and then subjected to the microscopic inspection. Cells of each transwell were counted in 8 random fields at 200 × magnification under Olympus microscope (Olympus Corporation, Japan).

### Microfluidic chip experiment

**Figure 13:**
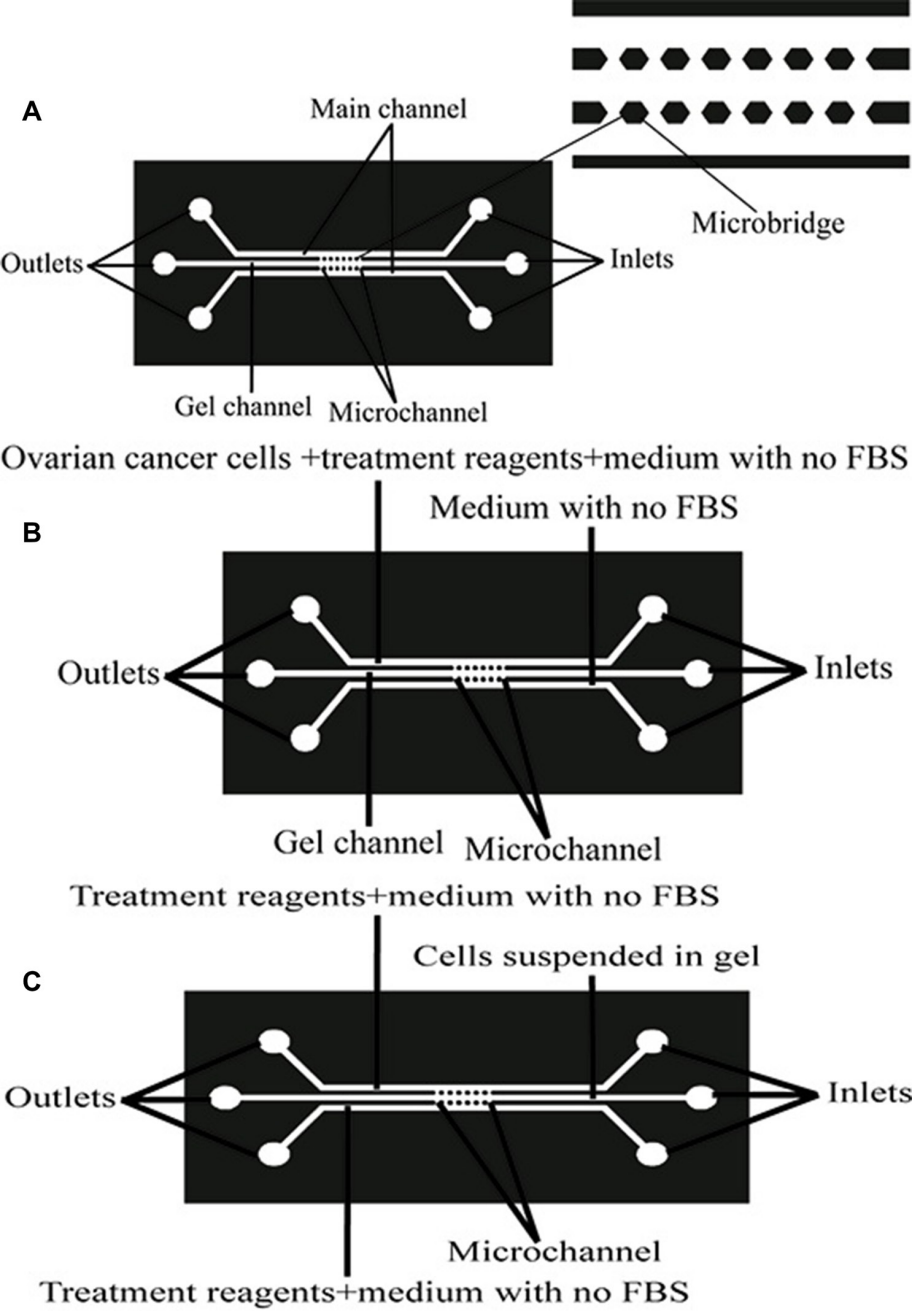
Design and structure of a microfluidic device for establishing an *in vitro* 3D cell growth and migration microenvironment model (**A**) Configuration of the device. The microfluidic device is composed of three main parallel channels connected by several smaller horizontal microchannels. (**B**) The schematic diagram of the microfluidic device for the migration assay of ovarian cancer cells. After matrigel was filled into the middle channel and polymerized, ovarian cancer cells were seeded into one of the side channels and treated them with setting conditions. The other side channel was filled with medium without FBS to determine the ability of cells to migrate into the gel. (**C**) The schematic diagram of the microfluidic device for cell colony formation in 3D conditions. Ovarian cells suspended in matrigel were injected into the middle channel, and both side channels were filled with condition medium to examine the ability of cell colony formation in the 3D gel.

We have successful experiences of the design and fabrication methods of microfluidic chip to go by Dai Xiaozhen, Cai Shaoxi, Qunfang Ye in our laboratory colleagues [163, 164]. Briefly, the microfluidic device is composed of three parallel main channels and several smaller horizontal regular hexagon microchannels which connect to the main channels, which can provide an *in vivo*-like microenvironment for the culture cells. Each main channel is 500 μm in width, 150 μm in height and 20 mm in length, while each bridge channel is 150 μm in width, 150 μm in height and 200 μm in length, the diameter of the circular holes both ends of the channel is 3 mm (Figure 13A). The matrigel (BD Biosciences, San Jose, CA, USA) was thawed overnight at 4°C on ice, and the pipettes, tips and microfluidic device were precooled at −20°C before use. In our experiments, it is divided into the following experimental groups: (1) The matrigel was mixed to homogeneity with cooled pipettes and injected into the middle channel of the microfluidic device to form a 3D model of the cell culture (Figure 13B). The cell suspensions were injected into one side of the microfluidic channel and allowed to adhere to the substrate for resuming the normal cell growth pattern under the 3D culture conditions. Then, the serum-free medium containing 0 ng/ml NGF, 100 ng/ml NGF, 100 ng/ml NGF+10 uM Ro 08-2750, 100 ng/ml NGF+100 uM K252a, 100 ng/ml NGF+5 nM LM11A-31 and 100 ng/ml NGF+100 uM K252a+5 nM LM11A-31 were respectively perfused into the channel after the cells were starved with serum-free medium for 24 hours. At the same time, the serum-free medium containing 0.1% BSA was injected into the other side of the channel to induce the cell migration (Figure 13B). (2) High density ovarian cancer cell suspensions (10^7^ cells/mL) were harvested and mixed with the thawed matrigel solution (2:1, v/v), then injected into the middle channel of the microfluidic device to mimic a relatively real 3D cell growth environment (Figure 13C). The serum-free medium containing 0 ng/ml NGF, 100 ng/ml NGF, 100 ng/ml NGF+10 uM Ro 08-2750, 100 ng/ml NGF+100 uM K252a, 100 ng/ml NGF+5 nM LM11A-31 and 100 ng/ml NGF+100 uM K252a+5 nM LM11A-31 were respectively perfused into the both sides of the channels after the Cell-Matrigel mixture was polymerized in order to assess the effects on colony formation of tumor cells under the 3D culture conditions (Figure 13C). The migration and colony formation were monitored by phase-contrast microscopy (Leica DM750, Wetzlar, Germany) after 24 hours with above reagents. The measurement of corresponding migration area and maximum migration distance was quantified by using Image J (http://rsbweb.nih.gov/ij/) [163, 165].

### Statistical analysis

Statistical analysis was performed applying the SPSS software (version PASW Statistics 18.0). Results are presented as mean ± sd from three independent experiments and the difference between them was analyzed by Student *t-test* when only 2 groups were compared, and the difference among them was analyzed by using ANOVA when 3 or more groups were compared. All *p*-values were considered statistically significant at *P* < 0.05, extremely significant at *P* < 0.01.

## SUPPLEMENTARY MATERIALS


