# Supplementary material for: Nerve growth factor modulates the tumor cells migration in ovarian cancer through the WNT/β-catenin pathway
Source: Oncotarget. 2016 Nov 7;7(49):81026–48. doi: 10.18632/oncotarget.13186 (PMC5348374; doi:10.18632/oncotarget.13186)
Supplement: Supplementary file 1 [file oncotarget-07-81026-s001.pdf]

Nerve growth factor modulates the tumor cells migration in ovarian cancer through the WNT/β-catenin pathway

Supplementary Materials

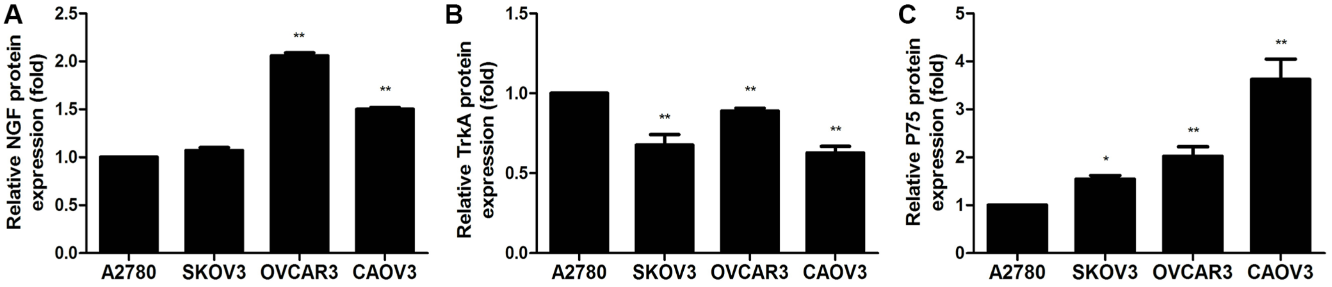

Supplementary Figure S1: NGF/NGFRs protein expression in human ovarian cancer cells. (**A–C**) NGF, TrkA and P75 protein expression in ovarian cancer cells measured by western blot with the indicated antibodies, and we had densitometric quantification of them with Quantity One soft. The value are expressed as mean ± sd. compared with A2780 (**$p < 0.01$). GAPDH protein expression served as a control for western blot. Data represent three replicate experiments, independently. (Figure 1 is corresponding to the original Figure 1).

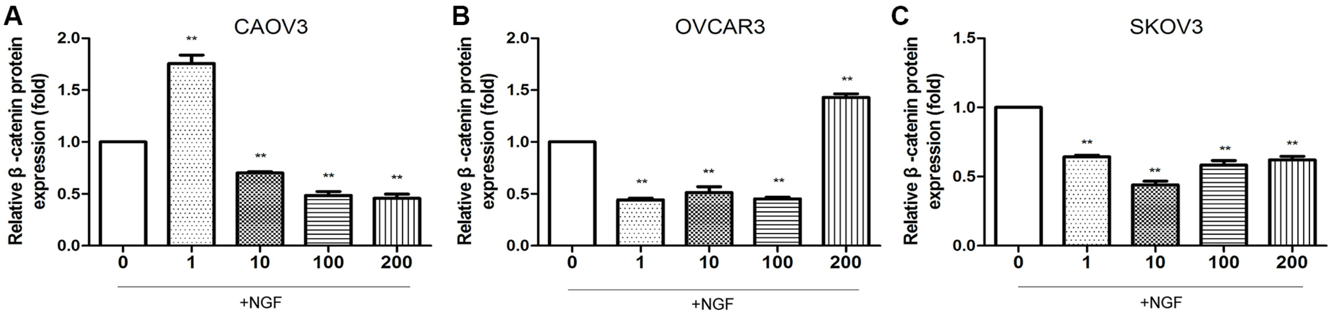

Supplementary Figure S2: The β-catenin protein expression in ovarian cancer cells stimulated with different concentrations of recombinant human β-NGF at 24 hour time point. (**A–C**) The β-catenin protein expression levels were analyzed in CAOV3, OVCAR3 and SKOV3 cells treated with different concentrations of recombinant human β-NGF (0–200 ng/ml) at 24 hour time point by western blot. GAPDH expression served as control. The value are expressed as mean ± sd. compared with the untreated control (0 ng/ml NGF, **$p < 0.01$.) Data represent three replicate experiments, independently. (Figure 2 is corresponding to the original Figure 3).

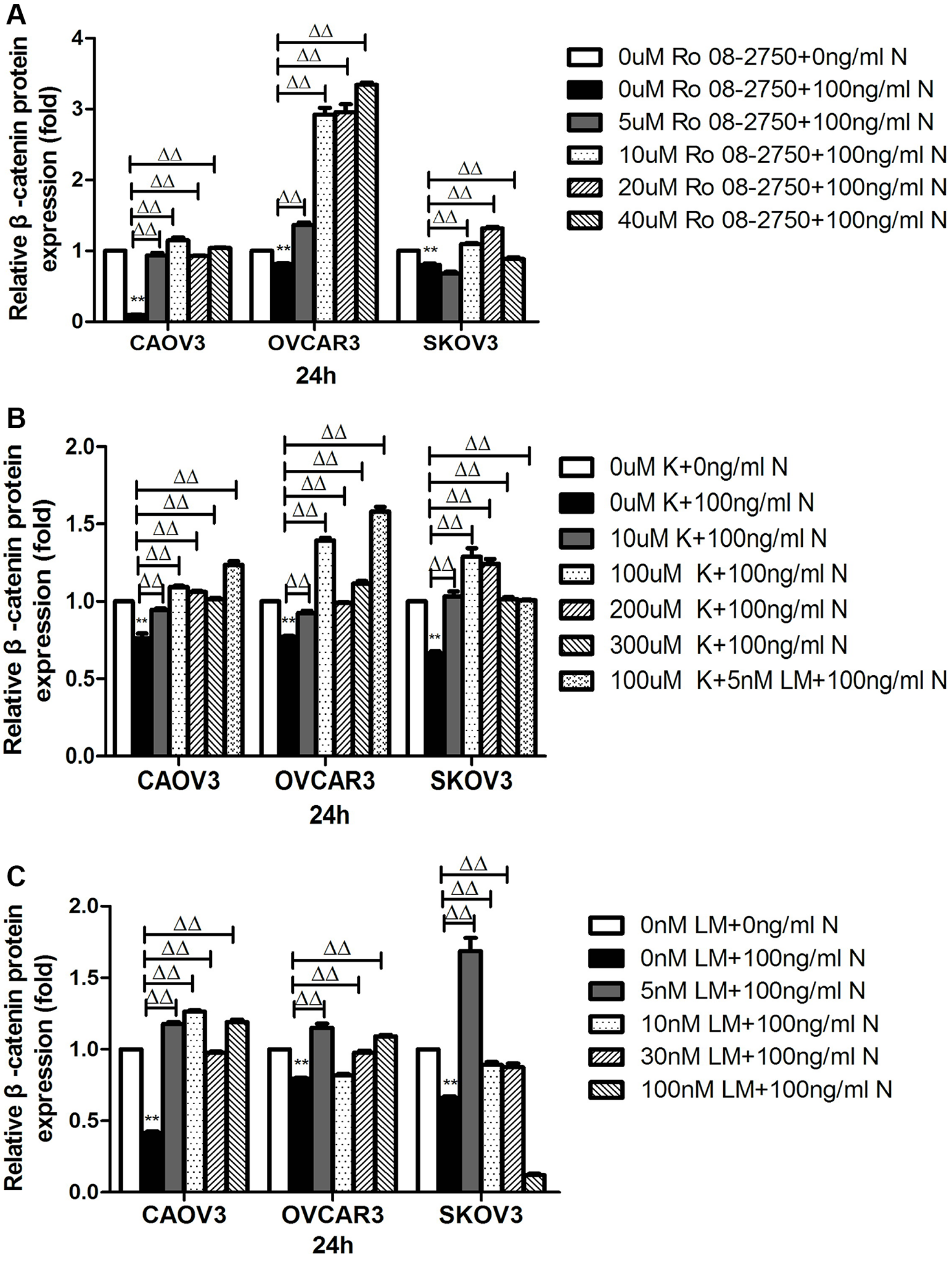

Supplementary Figure S3: The effect of NGF/NGFRs-related inhibitors on β-catenin protein expression in ovarian cancer cells at 24 hour time point. (**A**) The densitometric quantification of β-catenin protein expression with Quantity One soft. (Con: 0 uM Ro 08-2750+0 ng/ml NGF; 0: 0 uM Ro 08-2750+100 ng/ml NGF; 5: 5 uM Ro 08-2750+100 ng/ml NGF; 10: 10 uM Ro 08-2750+100 ng/ml NGF; 20: 20 uM Ro 08-2750+100 ng/ml NGF; 40: 40 uM Ro 08-2750+100 ng/ml NGF). (**B**) The densitometric quantification of β-catenin protein expression with Quantity One soft. (Con: 0 uM K252a+0 ng/ml NGF; 0: 0 uM K252a+100 ng/ml NGF; 10: 10 uM K252a+100 ng/ml NGF; 100: 100 uM K252a+100 ng/ml NGF; 200: 200 uM K252a+100 ng/ml NGF; 300:300 uMK252a+100 ng/ml NGF; 100K+5L: 100 uM K252a+5 nM LM11A-31+100 ng/ml NGF). (**C**) The densitometric quantification of β-catenin protein expression with Quantity One soft. (Con: 0 nM LM11A-31+0 ng/ml NGF; 0: 0 nM LM11A-31+100 ng/ml NGF; 5: 5nM LM11A-31+100 ng/ml NGF; 10: 10nM LM11A-31+100 ng/ml NGF; 30: 30 nM LM11A-31+100 ng/ml NGF; 100: 100 nM LM11A-31+100 ng/ml NGF). GAPDH expression served as control. The value are expressed as mean ± sd. compared with the untreated control (0 ng/ml NGF, $**p < 0.01$.) and 100 ng/ml NGF group ($\Delta\Delta p < 0.01$.) Data represent three replicate experiments, independently. (Figure 3 is corresponding to the original Figure 4).

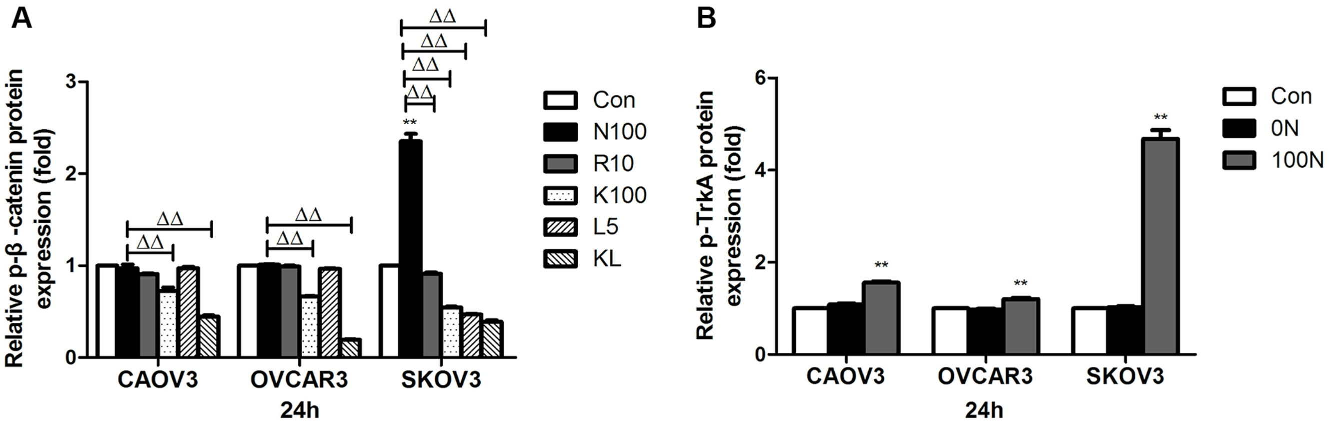

Supplementary Figure S4: Effect of NGF and inhibitors of NGF/NGFRs on β-catenin phosphorylation and TrkA phosphorylation in ovarian cancer cells. (**A**) The effect of 24 hour of treatment with NGF and the inhibitors of NGF/NGFRs on β-catenin phosphorylation in ovarian cancer cells (Con: non-stimulated, untreated ovarian cancer cells; N100: 100 ng/ml NGF; R10: 10 uM Ro 08-2750; K100: 100 uM K252a; L5: 5 nM LM11A-31; KL: 100 uM K252a+5 nM LM11A-31). The value are expressed as mean ± sd. compared with the untreated control (0 ng/ml NGF, $^{**}p < 0.01$.) and 100 ng/ml NGF group ($\Delta\Delta p < 0.01$.) (**B**) The TrkA phosphorylation in ovarian cancer cells under different culture conditions (Con: conventional culture condition; 0N: serum-free medium; 100N: 100 ng/ml NGF). The densitometric quantification were gained by Quantity One soft. The value are expressed as mean ± sd. compared with the conventional culture condition group ($^{**}p < 0.01$.) GAPDH expression served as control. Data represent three replicate experiments, independently. (Figure 4 is corresponding to the original Figure 6).

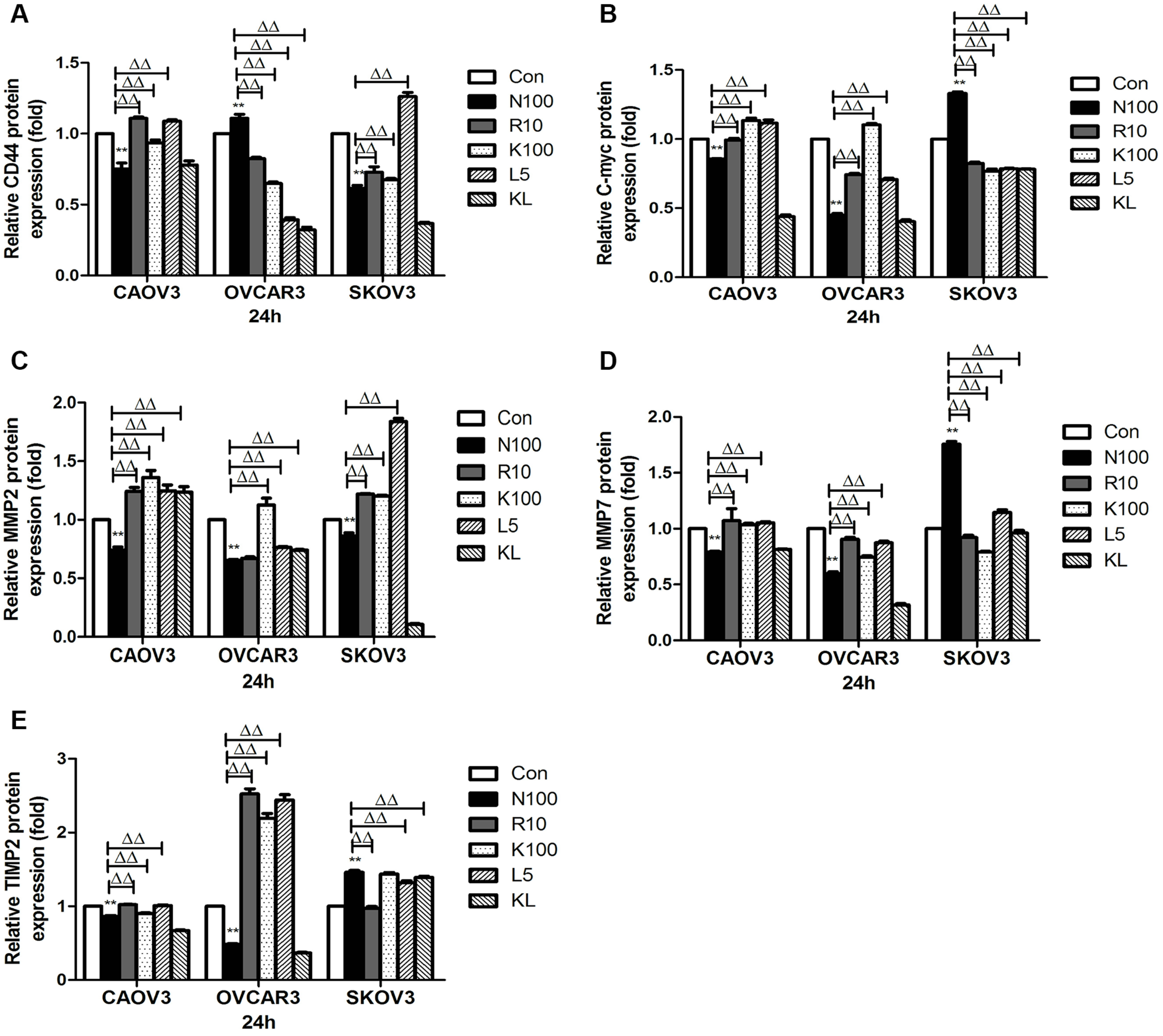

Supplementary Figure S5: NGF and related inhibitors of NGF/NGFRs modulate the protein expression levels of WNT/β-catenin downstream target genes on ovarian cancer cells. (**A**) The NGF and NGF/NGFRs-related inhibitors modulate the protein expression levels of CD44 in ovarian cancer cells at 24 hour time point. (**B**) The NGF and NGF/NGFRs-related inhibitors modulate the protein expression levels of C-myc in ovarian cancer cells at 24 hour time point. (**C**) The NGF and NGF/NGFRs-related inhibitor modulate the protein expression levels of MMP2 in ovarian cancer cells at 24 hour time point. (**D**) The NGF and NGF/NGFRs-related inhibitor modulate the protein expression levels of MMP7 in ovarian cancer cells at 24 hour time point. (**E**) The NGF and NGF/NGFRs-related inhibitor modulate the protein expression levels of TIMP2 in ovarian cancer cells at 24 hour time point. (100 ng/ml N: 100 ng/ml NGF; Con: 0 ng/ml NGF; N 100:100 ng/ml NGF; R10:10 uM Ro 08-2750+100 ng/ml NGF; K100:100 uM K252a+100 ng/ml NGF; L5:5 nM LM11A-31+100 ng/ml NGF; KL: 100 uM K252a+5 nM LM11A-31+100 ng/ml NGF). The value represent fold difference in relation to the untreated control (0 ng/ml NGF, $^{**}p < 0.01$.) and 100 ng/ml NGF group ($\Delta\Delta p < 0.01$.) Data represent three replicate studies and mean ± sd. GAPDH expression served as control in western blot assay. Data represent three replicate experiments, independently. (Figure 5 is corresponding to the original Figure 8).
